# Single-cell morphological and transcriptome analysis unveil inhibitors of polyploid giant breast cancer cells in vitro

**DOI:** 10.1038/s42003-023-05674-5

**Published:** 2023-12-21

**Authors:** Mengli Zhou, Yushu Ma, Chun-Cheng Chiang, Edwin C. Rock, Samuel Charles Butler, Rajiv Anne, Svetlana Yatsenko, Yinan Gong, Yu-Chih Chen

**Affiliations:** 1 UPMC Hillman Cancer Center, University of Pittsburgh, 5115 Centre Ave, Pittsburgh, PA 15232 USA; 2 Department of Computational and Systems Biology, University of Pittsburgh, 3420 Forbes Avenue, Pittsburgh, PA 15260 USA; 3 Xiangya Hospital, Central South University, Changsha, Hunan 410008 China; 4 Department of Bioengineering, Swanson School of Engineering, University of Pittsburgh, 3700 O’Hara Street, Pittsburgh, PA 15260 USA; 5 Department of Pathology, University of Pittsburgh, Pittsburgh, PA USA; 6 Department of Obstetrics, Gynecology and Reproductive Sciences, University of Pittsburgh, Pittsburgh, PA USA; 7 Magee Womens Research Institute, Pittsburgh, PA USA; 8 Department of Immunology, University of Pittsburgh School of Medicine, Pittsburgh, PA 15261 USA; 9 CMU-Pitt Ph.D. Program in Computational Biology, University of Pittsburgh, 3420 Forbes Avenue, Pittsburgh, PA 15260 USA

**Keywords:** Biological techniques, Cancer

## Abstract

Considerable evidence suggests that breast cancer therapeutic resistance and relapse can be driven by polyploid giant cancer cells (PGCCs). The number of PGCCs increases with the stages of disease and therapeutic stress. Given the importance of PGCCs, it remains challenging to eradicate them. To discover effective anti-PGCC compounds, there is an unmet need to rapidly distinguish compounds that kill non-PGCCs, PGCCs, or both. Here, we establish a single-cell morphological analysis pipeline with a high throughput and great precision to characterize dynamics of individual cells. In this manner, we screen a library to identify promising compounds that inhibit all cancer cells or only PGCCs (*e.g*., regulators of HDAC, proteasome, and ferroptosis). Additionally, we perform scRNA-Seq to reveal altered cell cycle, metabolism, and ferroptosis sensitivity in breast PGCCs. The combination of single-cell morphological and molecular investigation reveals promising anti-PGCC strategies for breast cancer treatment and other malignancies.

## Abstract (web summary)

Breast cancer resistance and relapse may stem from polyploid giant cancer cells (PGCCs), and eradicating them poses a challenge. A precise, high-throughput single-cell analysis can identify compounds that target non-PGCCs, PGCCs, or both cell types.

## Introduction

At 2.3 million new cases, breast cancer was the most diagnosed cancer in 2020. It represents 11.7% of cancer diagnoses and is the leading cause of cancer death in women, accounting for 1 in 6 cancer deaths^1^. Despite the achievements made in introducing new treatment strategies in the past decades, the development of tumors toward therapy resistance is prevalent^2,3^. Many treatment resistance mechanisms of breast cancer were reported, and growing evidence suggests the resistance can be driven by polyploid giant cancer cells (PGCCs)^4–6^. PGCCs are a special sub-population of cancer cells with multiple nuclei or a single giant nucleus containing multiple sets of chromosomes^7^. While polyploidy is a normal physiological process in the development of cardiomyocytes and many other cell types^8,9^, the mechanism was hijacked by cancer cells to enhance their resistance to therapies^10,11^. Given that polyploid cells exist in premalignant tissues, the number of PGCCs increases with progression of disease and therapeutic stress^12–16^. In addition, oncoviruses and cytomegalovirus trigger the appearance of PGCCs in tumors^17–19^. As compared to non-PGCCs, PGCCs are more resistant to chemotherapy. Once treatment stress is relieved, relapse from PGCCs by budding to non-PGCC has been observed^20^. Both clinical observations and in vitro experiments highlight the importance of PGCCs in breast cancer therapeutic resistance and relapse as well as other solid tumor malignancies.

It was reported that PGCCs acquire the properties of cancer stem-like cells (CSCs), supported by their enhanced tumor-initiating capability and up-regulation of relevant biomarkers, including octamer-binding transcription factor-4 (OCT4), NANOG, sex-determining region Y-box 2 (SOX2), aldehyde dehydrogenase-1A (ALDH1A), CD44, and CD133^21–23^. In addition, PGCCs were found to correlate with epithelial-to-mesenchymal transition (EMT) by elevated cell motility, overexpression of hypoxia-inducible factor-1α (HIF-1α), Twist, Snail, Zinc Finger E-Box Binding Homeobox 2 (ZEB2), Vimentin, N-cadherin, and Fibronectin, and down-regulation of E-cadherin^5,15,21,24,25^. The association with tumor initiation and EMT further strengthens the clinical value to inhibit this tumor sub-population. Recently, using sequencing data from ~10,000 primary human cancer samples and ~600 cancer cell lines, Whole genome doubling (WGD) cells were found to depend on spindle assembly checkpoint signaling, DNA replication factors, and proteasome function^4,26^.

Researchers have explored anti-PGCC strategies and unveiled immunosurveillance against cancer cell polyploidy^27–30^. However, so far, there is no effective anti-PGCC therapy^4^. The delay in developing anti-PGCC therapies is caused by the lack of a high-throughput method to rapidly quantify PGCCs. Conventional drug screening assays (e.g., MTT, XTT, or ATP) rapidly measure the overall inhibition of bulk cancer cells yet provide little information about the eradication of a small number of PGCCs, which can cause treatment resistance and relapse. As PGCCs are defined by their excess DNA content as well as large cell and nuclear size, the current gold standard to identify/isolate PGCCs is based on fluorescence-activated cell sorting (FACS) and visual confirmation^21^. While flow cytometry can quantify the number and percentage of PGCCs, it is impractical to (1) screen hundreds or thousands of compounds using flow cytometry or (2) monitor the dynamic process of PGCC induction and death. Image-based segmentation and detection of cells have been developed, but the methods have not been widely used in the studies of PGCCs^31–33^. The limitations of existing approaches highlight the need to establish a high-throughput and precise analytical method for PGCC studies.

**Fig. 1:**
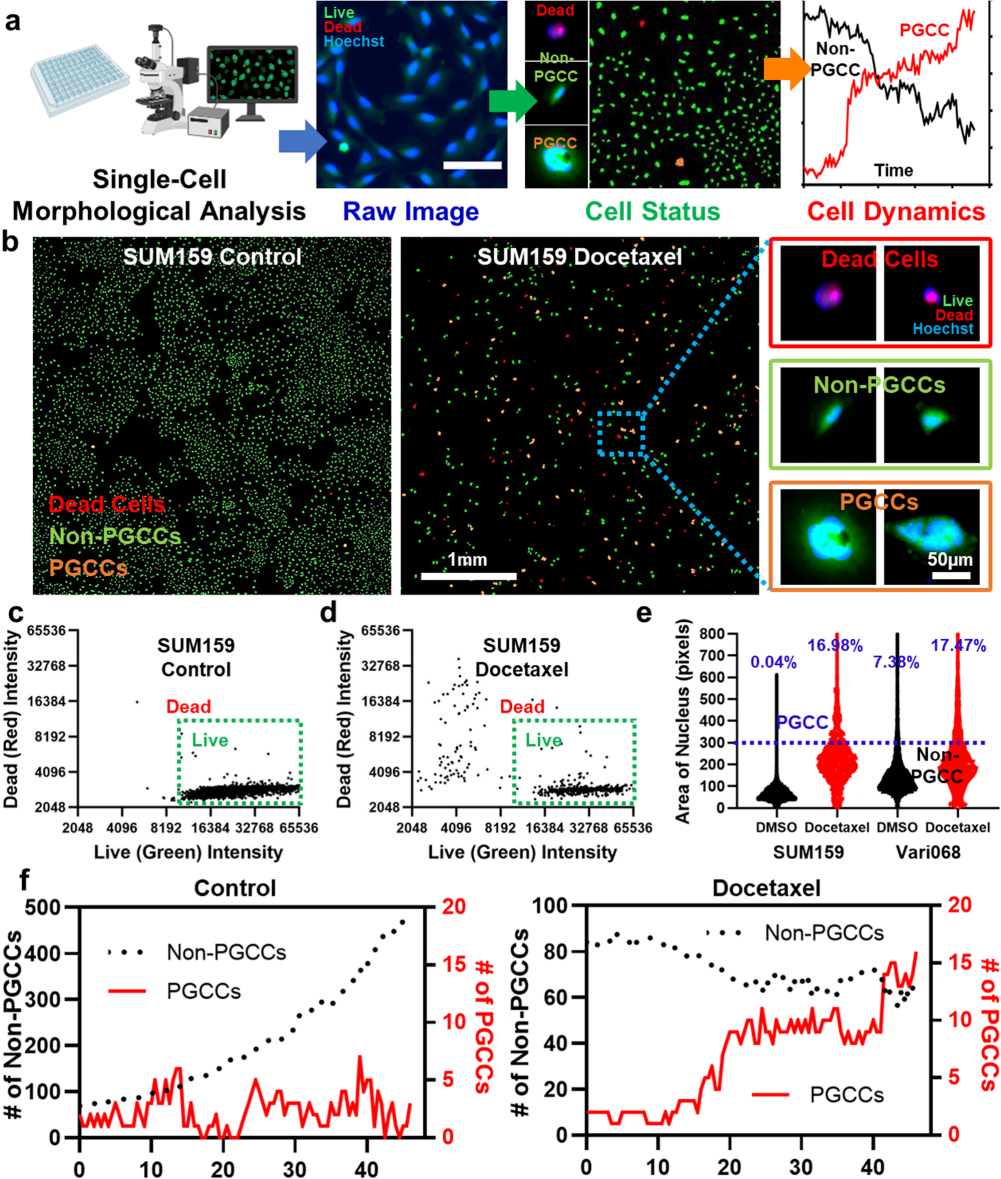
Single-cell morphological analysis to identify PGCCs. **a** A conceptual diagram of single-cell morphological analysis, which can rapidly identify individual cells as PGCCs, non-PGCCs, and dead cells and monitor the dynamics of cell populations. The diagram was generated with elements from the Biorender. **b** The single-cell morphological analysis of control and Docetaxel-treated SUM159 cells. Without treatment, 5129 non-PGCCs, 2 PGCCs, and 20 dead cells were recognized. With Docetaxel treatment, the population was shifted to 356 non-PGCCs, 76 PGCCs, and 124 dead cells. The green dots represent non-PGCCs, the orange dots represent PGCCs, and the red dots represent dead cells (Scale bar: 1 mm). The enlarged images of representative SUM159 breast cancer PGCCs, non-PGCCs, and dead cells are also presented (Scale bar: 50 μm). **c**, **d** Separation of live and dead SUM159 cells by Live/Dead staining using the single-cell morphological analysis. Each dot represents a cell. The *x*-axis represents the fluorescent intensity of Live (FITC green) staining, and the *y*-axis represents the fluorescent intensity of Dead (TRITC red) staining. Only cells with high Live intensity and low Dead intensity were considered live cells. **c** DMSO control (*n* = 5685 cells). **d** 1 μM Docetaxel treatment (*n* = 566 cells). **e** Identification of PGCCs and non-PGCCs by single-cell morphological analysis according to the area of cell nuclei. SUM159 and Vari068 breast cancer cells were treated with DMSO control (5674 non-PGCCs and 2 PGCCs for SUM159 and 2,059 non-PGCCs and 164 PGCCs for Vari068) and 1 μM Docetaxel (396 non-PGCCs and 81 PGCCs for SUM159 and 841 non-PGCCs and 178 PGCCs for Vari068). **f** Dynamics of cell status. SUM159 cells with and without Docetaxel treatment were imaged every 30 min for 2 days. The *x*-axis represents time (hours), the left *y*-axis (black) represents the number of non-PGCCs, and the right *y*-axis (red) represents the number of PGCCs. The black curve indicates the number of non-PGCCs, and the red curve indicates the number of PGCCs. (*n* = 4).

Advances in modern imaging technology and computer vision provide an attractive alternative to quantify PGCCs by cellular morphological analysis. In this work, we established a high-throughput, reliable, and precise single-cell morphological analysis pipeline to rapidly quantify the number of PGCCs and non-PGCCs (Fig. 1). In addition to a snapshot one-time measurement, the dynamics of PGCC development can be monitored. The method can be widely applied to PGCC studies, especially screening for anti-PGCC compounds. Using our innovative method, we screened a library of 172 regulators of cancer-relevant pathways. The experiments identified promising compounds that either inhibited all cancer cells or only PGCCs, including HDAC inhibitors, proteasome inhibitors, and ferroptosis inducers. In addition, we performed single-cell transcriptome sequencing (scRNA-Seq) to identify breast PGCCs’ unique molecular features. The altered cell cycle, metabolism, sensitivity to ferroptosis, and pathways of PLK1, Aurora, and FOXM1 suggest treatment strategies. This preliminary success provides not only promising compounds to kill PGCCs but also an innovative pipeline to investigate other heterogeneous cancer cell populations with high throughput.

## Methods

### Cell culture

We cultured MDA-MB-231, MDA-MB-436, MDA-MB-468, Vari068, and BT474 cells in Dulbecco’s modified eagle medium (DMEM, Gibco 11995) supplemented with 10% fetal bovine serum (FBS, Gibco 16000), 1% GlutaMax (Gibco 35050), 1% penicillin/streptomycin (pen/strep, Gibco 15070), and 0.1% of plasmocin (InvivoGen ant-mpp). We cultured SUM149 and SUM159 cells in F-12 (Gibco 11765) media supplemented with 5% FBS (Gibco 16000), 1% pen/strep (Gibco 15070), 1% GlutaMax (Gibco 35050), 1 μg mL^−1^ hydrocortisone (Sigma H4001), and 5 μg mL^−1^ insulin (Sigma I6634), and 0.1% of plasmocin (InvivoGen, ant-mpp). We cultured SKBR3 and T47D cells in RPMI 1640 medium (RPMI, Gibco 11875) supplemented with 10% fetal bovine serum (FBS, Gibco 16000), 1% GlutaMax (Gibco 35050), 1% penicillin/streptomycin (pen/strep, Gibco 15070), and 0.1% of plasmocin (InvivoGen, ant-mpp). The summary of breast cancer cell types is in Supplementary Table 1. MDA-MB-231, SUM149, and SUM159 cells were obtained from Dr. Gary Luker’s lab at the University of Michigan. Vari068, MDA-MB-436, MDA-MB-468, BT474, SKBR3, and T47D cells were obtained from Dr. Max Wicha’s lab at the University of Michigan. Vari068 is a patient-derived cell line (originally derived from an ER-/PR-/Her2- breast cancer patient who had signed informed consent) adapted to the standard two-dimensional culture environment^34–36^. We maintained all cells at 37 °C in a humidified incubator with 5% CO_2_. All the cells were cultured and passaged when the cells reached over 80% confluency in the dish. Cell lines have been authenticated by short tandem repeat profiling. All cell lines were cultured with mycoplasma antibiotics Plasmocin and examined for mycoplasma contamination by sensitive PCR assays.

### Cell transfection

We stably transfected TNBC cells using Xfect™ Transfection Reagent (Takara 631317) with 5 µg of pDsRed2-Nuc Vector plasmid (Takara 632408). The red fluorescent proteins (DsRed2) fused with three copies of the nuclear localization signal (NLS) of the simian virus 40 large T-antigen translocated into the nucleus of cancer cells, so nucleus-red cells could be generated for time-lapse tracking of population dynamics. The transfected cells were selected using G418 (Takara 631307) treatment and sorted by flow cytometry for red fluorescence.

### Compound screening to identify inhibitors of PGCCs

**Fig. 2:**
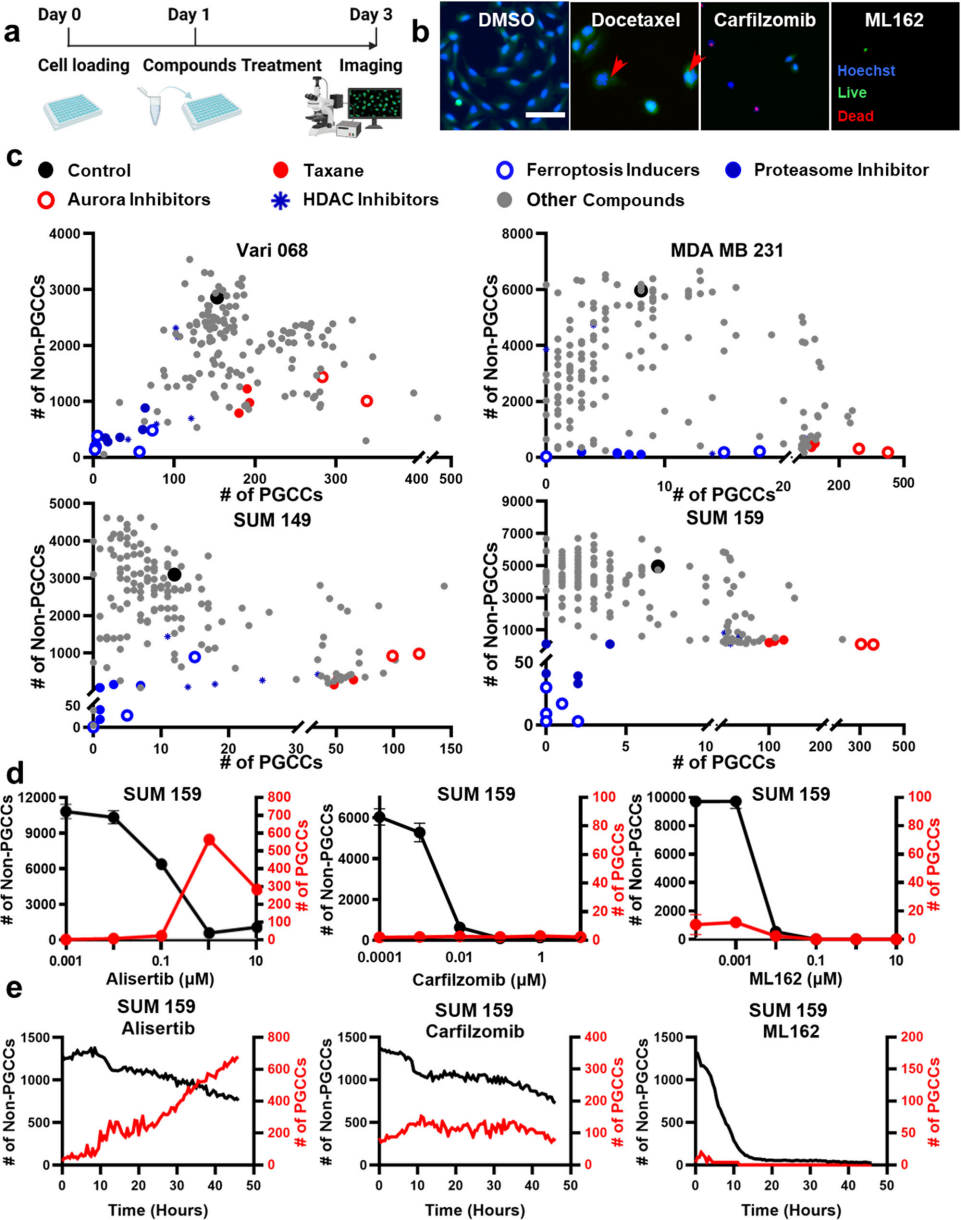
Comprehensive analysis of compound efficacy by quantifying PGCCs and non-PGCCs. **a** Workflow of the compound screening experiments. Day 0: cell loading; Day 1: compounds treatment; Day 3: Live/Dead/Hoechst staining and imaging for single-cell morphological analysis. The diagram was generated with elements from the Biorender. **b** Representative images of SUM159 treated with DMSO control, 1 μM Docetaxel, Carfilzomib, and ML162. Cells were stained with Live (green), Dead (red) and Hoechst (blue) staining reagents. PGCCs are marked with red arrows. (Scale bar: 100 μm) **c** Screening the effects of 159 compounds using Vari068, MDA-MB-231, SUM149 and SUM159 breast cancer cells. The *x*-axis represents the number of PGCCs after treatment, and the *y*-axis represents the number of non-PGCCs. Each dot represents the treatment of a compound. Different colors are used to indicate different classes of compounds. Statistical analysis is provided in Supplementary Data 2 and 3. **d** Treatments of selected compounds (Alisertib, Carfilzomib, and ML162) on SUM159 cells. The *x*-axis represents the concentration of the compound. The left *y*-axis (black) represents the number of non-PGCCs, and the right *y-*axis (red) represents the number of PGCCs. The black curve indicates the number of non-PGCCs, and the red curve indicates the number of PGCCs. Error bars indicate the standard error of the mean (SEM), *n* = 3. **e** Dynamics of cell status. SUM159 cells treated with Alisertib, Carfilzomib, and ML162 were imaged every 30 min for 2 days. The *x*-axis represents time (hours), the left *y*-axis (black) represents the number of non-PGCCs, and the right *y*-axis (red) represents the number of PGCCs. The black curve indicates the number of non-PGCCs, and the red curve indicates the number of PGCCs. (*n* = 4).

**Fig. 3:**
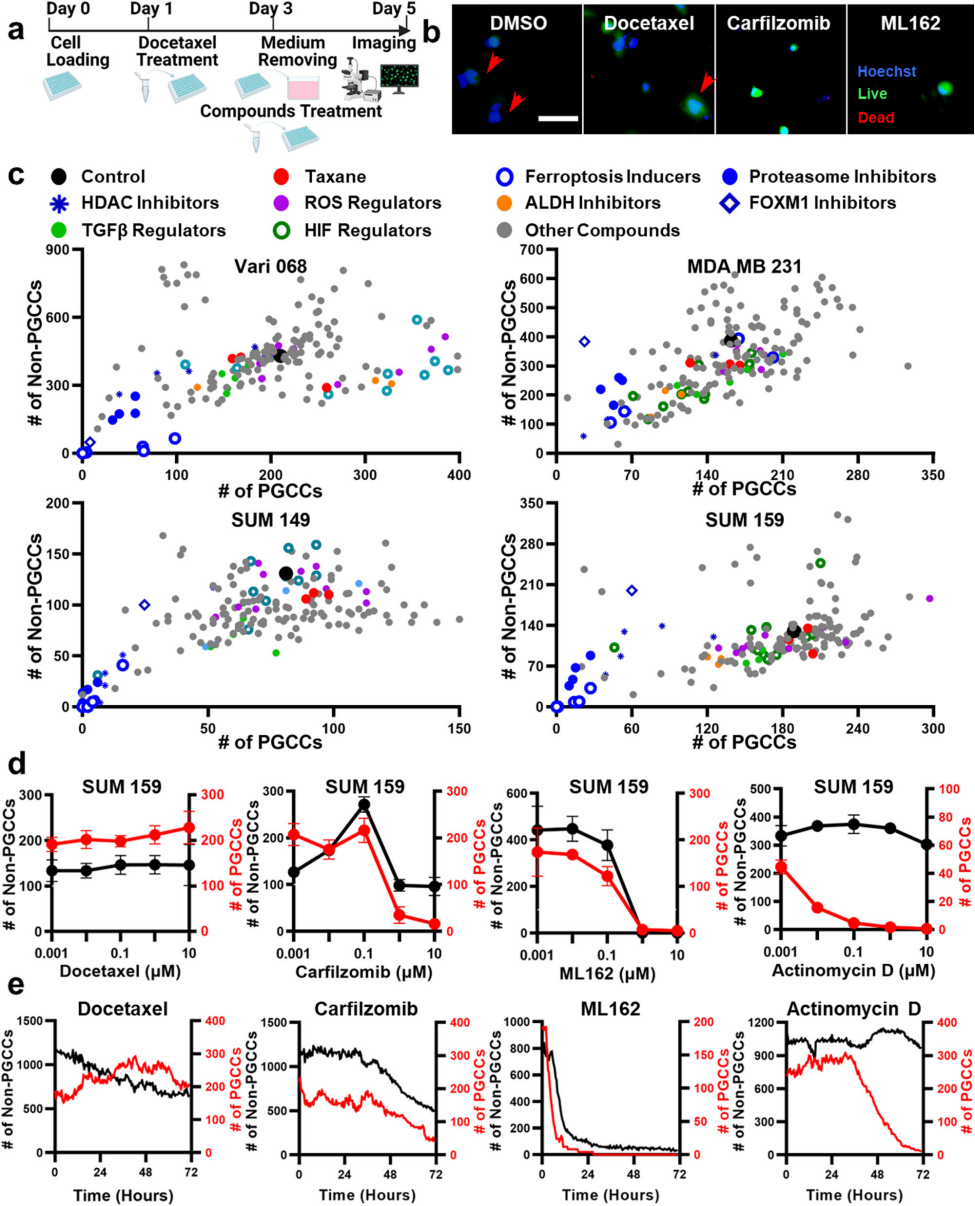
Discover anti-PGCC compounds by screening experiments. **a** Workflow to discover anti-PGCC compounds. Day 0: cell loading; Day 1: induction of PGCCs by 1 μM Docetaxel treatment; Day 3: removal of supernatant and screening of a library of 172 compounds; Day 5: staining and imaging cells for single-cell morphological analysis. The diagram was generated with elements from the Biorender. **b** Representative images of SUM159 treated with DMSO control, 1 μM Docetaxel, Carfilzomib, and ML162. Cells were stained with Live (green), Dead (red), and Hoechst (blue) staining reagents. PGCCs are marked with red arrows. (Scale bar: 100 μm). **c** Screening the effects of 172 compounds using Vari068, MDA-MB-231, SUM149, and SUM159 breast cancer cells. The *x*-axis represents the number of PGCCs after treatment, and the *y*-axis represents the number of non-PGCCs. Each dot represents the treatment of a compound. Different colors are used to indicate different classes of compounds. Statistical analysis is provided in Supplementary Data 4, 5. **d** Treatments of selected compounds (Docetaxel, ML162, Carfilzomib, and Actinomycin D) at 5 concentrations (10 μM, 1 μM, 0.1 μM, 0,01 μM, and 0.001 μM) on SUM159 cells. The *x*-axis represents the concentration of the selected compound. The left *y*-axis (black) represents the number of non-PGCCs, and the right *y*-axis (red) represents the number of PGCCs. The black curve indicates the number of non-PGCCs, and the red curve indicates the number of PGCCs. Error bars indicate the standard error of the mean (SEM), *n* = 3. **e** Dynamics of cell status. SUM159 cells pre-treated with Docetaxel for 2 days to induce PGCCs were treated with Docetaxel, ML162, Carfilzomib, and Actinomycin D and imaged every 30 min for 3 days. The *x*-axis represents time (hours), the left *y*-axis (black) represents the number of non-PGCCs, and the right *y*-axis (red) represents the number of PGCCs. The black curve indicates the number of non-PGCCs, and the red curve indicates the number of PGCCs. (*n* = 4).

**Fig. 4:**
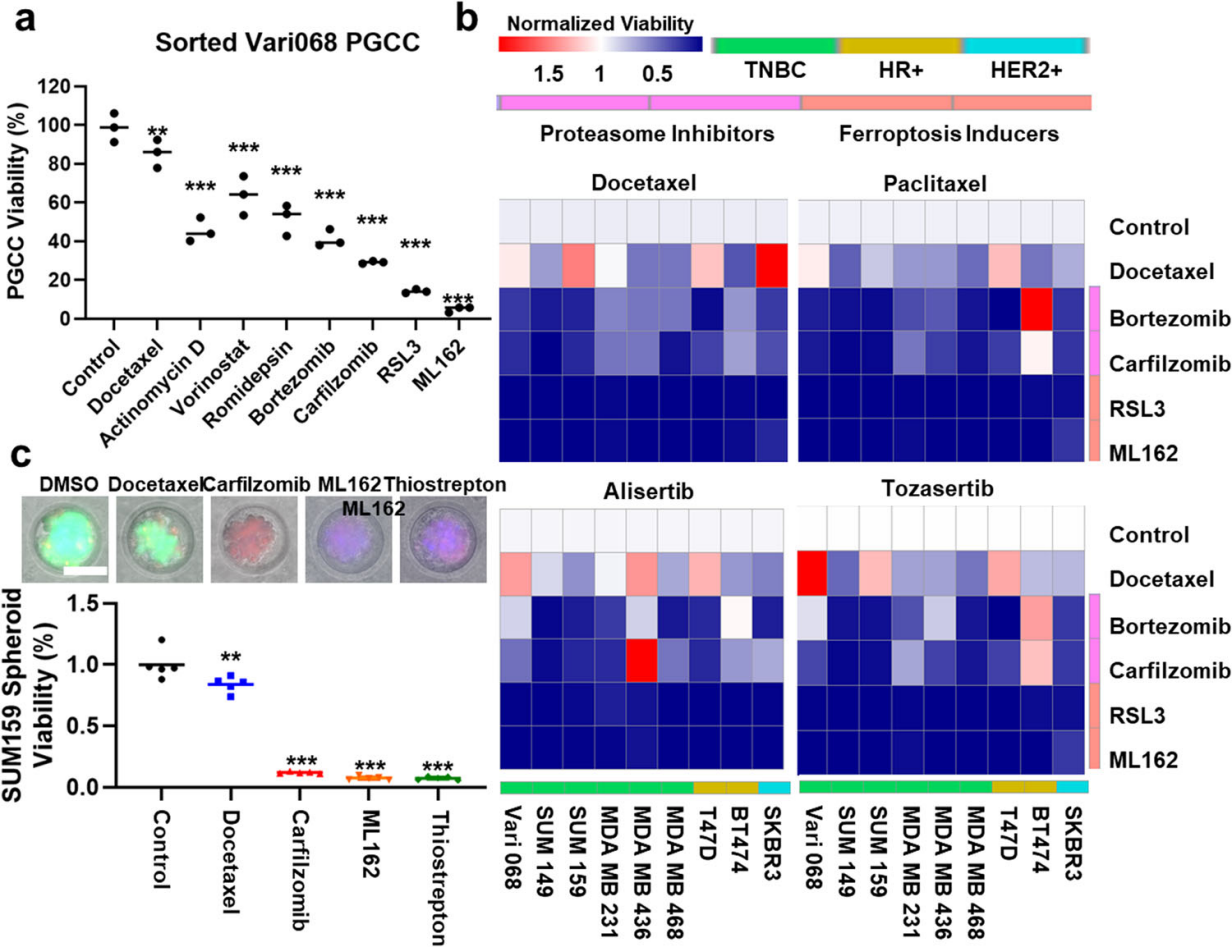
Validation of anti-PGCC compounds using additional cell lines, untreated PGCCs, and different PGCC-inducing methods. **a** Flow cytometry sorted untreated Vari068 PGCCs were treated by DMSO control, 1 μM Docetaxel, Actinomycin D, HDAC inhibitors (Vorinostat SAHA and Romidepsin), proteasome inhibitors (Bortezomib and Carfilzomib), and ferroptosis inducers (RSL3 and ML162). The *y*-axis represents the number of PGCCs after treatment of 2 days. Error bars indicate the standard error of the mean (SEM), *n* = 3. ** refers to *P* < 0.01, and *** refers to *P* < 0.001. **b** Inhibition of PGCCs induced by various methods: two chemotherapeutics (1 μM Docetaxel and Paclitaxel) and two Aurora inhibitors (1 μM Alisertib and Tozasertib). Nine breast cancer cell lines (HR-positive (T47D and BT474), HER2-positive (SKBR3), and TNBC (Vari068, SUM149, SUM159, MDA-MB-231, MDA-MB-436, and MDA-MB-468) were tested. Color gradient represents the cell viability normalized to the control. PGCCs generated by different methods and different cell lines were resistant to Docetaxel yet responded to proteasome inhibitors and ferroptosis inducers. Statistical analysis is provided in Supplementary Tables 2–5. **c** Drug testing on 3D cancer spheroid model. The consecutive treatments of Docetaxel and then PGCC-inhibiting compounds are significantly more effective than continuous treatment of Docetaxel and control. Error bars indicate the standard error of the mean (SEM), *n* = 5. ** refers to *P* < 0.01 and *** refers to *P* < 0.001.

**Fig. 5:**
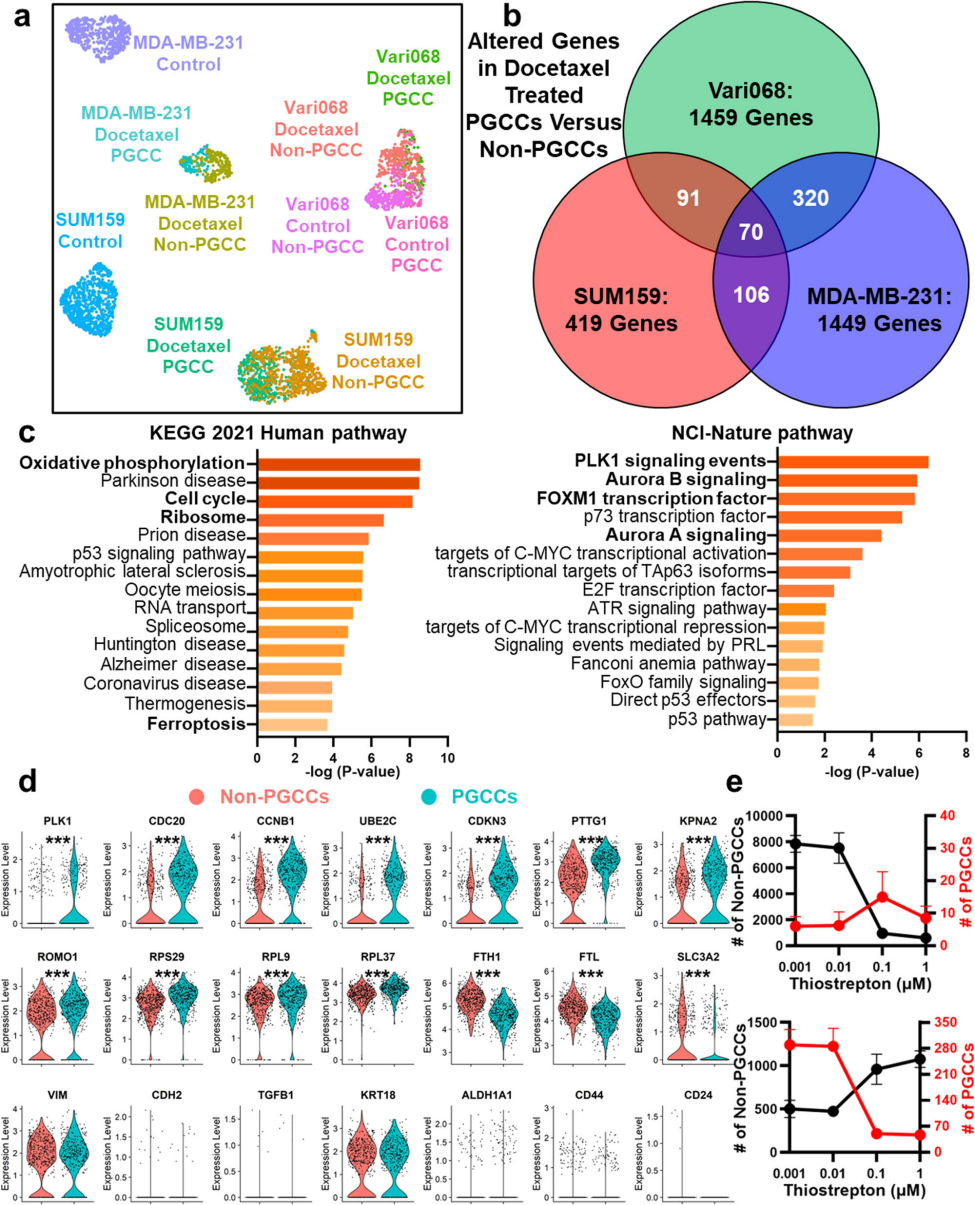
Single-cell transcriptome analysis of breast cancer PGCCs and non-PGCCs. **a** UMAP plot of single-cell transcriptome analysis, including PGCCs and non-PGCCs with and without Docetaxel treatment of three breast cancer cell lines (SUM159, MDA-MB-231, and Vari068). The *x*-axis represents UMAP1, the *y*-axis represents UMAP2, and each dot represents one cell. Different colors represent different cell populations/treatment conditions. Number of cells in each population were provided in the “Methods” section. **b** Comparison between altered genes in docetaxel-treated PGCCs vs. non-PGCCs of three breast cancer cell lines. **c** Top-ranked pathways (the KEGG 2021 Human and the NCI-Nature pathway databases) were determined by the altered genes of SUM159 PGCCs versus non-PGCCs. The *x*-axis and color represent the *P*-values, and the *y*-axis indicates the names of pathways. **d** Violin plots of docetaxel-treated SUM159 PGCCs and non-PGCCs cells with statistical tests. The *y*-axis represents gene expression with a logarithmic scale. Each dot represents one cell. * refers to *P* < 0.05. ** refers to *P* < 0.01, and *** refers to *P* < 0.001. **e** Treatment of Thiostrepton (FOXM1 inhibitor) at 4 concentrations (1 μM, 0.1 μM, 0,01 μM, and 0.001 μM) on SUM159 cells. The upper figure shows the treatment of cells without Docetaxel pre-treatment, and the lower figure shows the treatment of cells after Docetaxel pre-treatment. The *x*-axis represents the concentration of Thiostrepton. The left *y*-axis (black) represents the number of non-PGCCs, and the right *y*-axis (red) represents the number of PGCCs. The black curve indicates the number of non-PGCCs, and the red curve indicates the number of PGCCs. Error bars indicate the standard error of the mean (SEM), *n* = 4.

**Fig. 6:**
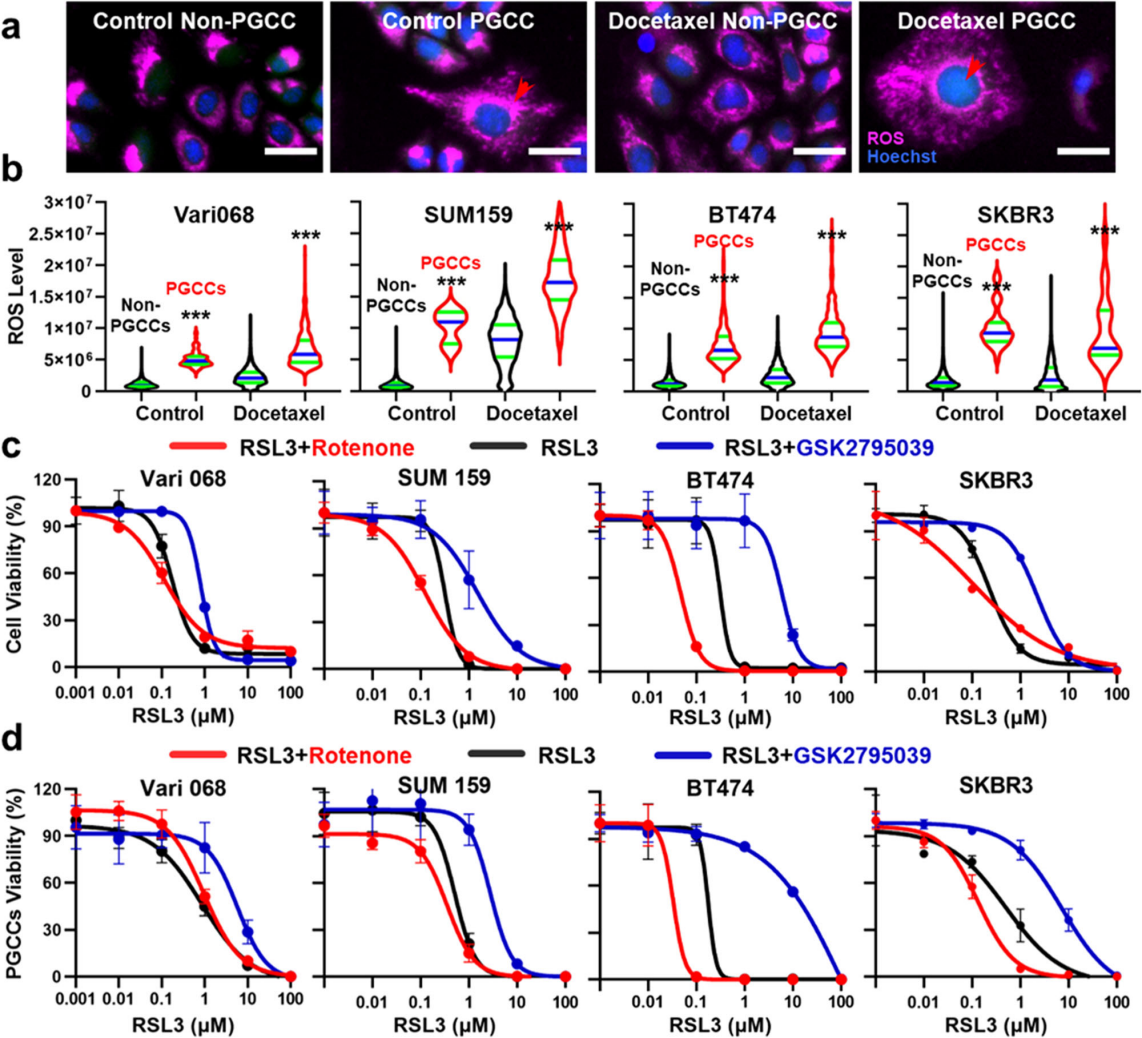
The elevated ROS level of PGCCs is essential to ferroptosis sensitivity. **a** Representative images of Vari068 PGCCs and non-PGCCs treated with DMSO control and 1 μM Docetaxel. Cells were stained with Live (green), Dead (red), Hoechst (blue), and CellROX™ Deep Red (Purple) staining. PGCCs are marked with red arrows. (Scale bar: 100 μm). **b** The ROS level of PGCC groups is significantly higher than non-PGCCs groups both for DMSO control and 1 μM Docetaxel treatment for Vari068 (Control: *n* = 16,082 non-PGCCs and 89 PGCCs, Docetaxel: *n* = 8455 non-PGCCs and 401 PGCCs), SUM159 (Control: *n* = 31,705 non-PGCCs and 6 PGCCs, Docetaxel: *n* = 1588 non-PGCCs and 133 PGCCs), BT474 (Control: *n* = 9181 non-PGCCs and 585 PGCCs, Docetaxel: *n* = 3885 non-PGCCs and 346 PGCCs) and SKBR3 (Control: *n* = 11,071 non-PGCCs and 39 PGCCs, Docetaxel: *n* = 3771 non-PGCCs and 18 PGCCs). The black violin plot indicates non-PGCCs, and the red violin plot indicates PGCCs. The green lines represent quartiles and the blue lines the median of all cells. **c** Rescue and sensitize cells to ferroptosis by altering the ROS level. Rotenone was selected to boost and GSK2795039 to reduce the ROS level. Compound efficacy IC50 curves of RSL3 (from 100 μM to 0.001 μM) alone (black), RSL3 with Rotenone (red), and RSL3 with GSK2795039 (blue) were tested for comparison. IC50s are provided in Supplementary Table 27. Error bars indicate the standard error of the mean (SEM), *n* = 3. **d** Rescue and sensitize induced PGCCs to ferroptosis by altering the ROS level. Docetaxel (1 μM) was treated to induce PGCCs for Vari068 and SUM159, and Alisertib (1 μM) was treated to induce PGCCs for BT474 and SKBR3 cell lines. Compound efficacy IC50 curves of RSL3 (from 100 μM to 0.001 μM) alone (black), RSL3 with Rotenone (red), and RSL3 with GSK2795039 (blue) were tested for comparison. IC50s are provided in Supplementary Tables 28 and 29. Error bars indicate the standard error of the mean (SEM), *n* = 3.

For our screening experiments, we assembled a library comprising 172 compounds, as detailed in Supplementary Data 1. These compounds were prepared at a concentration of 10 mM, dissolved in either DMSO or PBS, following the precise guidelines provided by the respective vendors. To facilitate the screening process, we subjected these compound solutions to serial dilution, ultimately achieving a final concentration of 1 µM for utilization in our screening experiments (Figs. 1–3). The application of DMSO at a concentration of 0.01% served as our control treatment. In preparation for the screening experiments, cells were harvested from culture dishes using 0.05% Trypsin/EDTA (Gibco 25200). Following this, the cells were gently centrifuged at 1,000 rpm for 4 min, subsequently re-suspended in the appropriate cell culture media, and seeded into the wells of 96-well plates. For the experiments of Figs. 2c, 4b, 5e, 6c, and Supplementary Figs. 5 and 6, depending on the proliferation rates of cell lines, different numbers of cells were seeded. For Vari068, SKBR3, MDA-MB-436, MDA-MB-468, T47D, and BT474 cells, 4000 cells in 100 μL media were seeded per well. For SUM149 cells, 2,000 cells in 100 μL media were seeded per well. For SUM159 and MDA-MB-231 cells, 1000 cells in 100 μL media were seeded per well. Following seeding, the cells underwent a 24-h cultivation period before being subjected to the compounds for a 48-h treatment duration. Upon completion of the treatment phase, the cells were stained using a combination of 0.8 μM Calcein AM, 1.6 μM Ethidium homodimer-1 (from the Invitrogen™ L3224 Live/Dead Viability/Cytotoxicity Kit), and 5 μM Hoechst 33342 (Thermo Scientific 62249). The staining conducted over a 30-min incubation period within the controlled environment of the incubator prepared the cells for subsequent imaging and analysis. For the experiments presented in Fig. 4a, Vari068 cells were flow-sorted to isolate PGCCs. We then seeded 4,000 sorted PGCCs in 100 μL of media per well for drug treatment. In the experiments corresponding to Figs. 3c and 6d, and Supplementary Figs. 7–15, 4000 cells were seeded in 100 μL of media per well for all cell lines. After 24 h of culture, the cells were exposed to PGCC-inducing agents for 48 h. Following this, the cell suspension was aspirated to eliminate the PGCC-inducing agents, and the testing compounds were directly introduced to treat mixed populations for an additional 48 h, without the need for flow sorting. The same staining and imaging protocol was applied to quantify the number of PGCCs and non-PGCCs following the treatments. We acknowledge that varying treatment timelines can substantially impact PGCC formation and survival. In this study, we have selected the treatment schedule as presented. In future investigations, we will explore the effects of different treatment schedules. For the experiments regulating cellular ROS level (Fig. 6), Rotenone (10 μM for Vari068 and SKBR3, 300 nM for SUM159, and 3 μM for BT474) was used to boost the ROS level, and GSK2795039 (40 μM for Vari068, BT474, and SKBR3 and 100 μM for SUM159) was used to reduce cellular ROS level. CellROX™ Deep Red Reagent (Thermo Scientific C10422) 5 μM was added for measuring the ROS level.

### Image acquisition

Cells in 96-well plates were imaged using an inverted microscope (Nikon Ti2E). Brightfield and fluorescence images were captured with a 4x or 10x objective lens and a monochrome CMOS camera (Hamamatsu ORCA-Fusion Gen-III SCMOS Camera). The field of view spans approximately 14 mm², accommodating up to 10,000 cells per image. Hoechst-stained cell nuclei were imaged via a DAPI filter set, while live and dead staining used FITC and TRITC filter sets, respectively. CellROX™ Deep Red staining was imaged using a Cy5 filter set. Auto-focusing ensured image clarity during imaging, taking under 9 min to capture images from a 96-well plate. Time-lapse experiments, tracking cell population dynamics, were conducted using a Tokai Hit stage top environment control on the Nikon Ti2E microscope.

### Single-cell morphological analysis software

The goal of image processing is to quantify the number of viable cells and distinguish PGCCs from non-PGCCs. A custom MATLAB (2021b) program was developed to perform the task in three steps: (1) identify cell nuclei with Hoechst staining, (2) determine whether the cell is alive or dead, (3) recognize PGCCs with the large size of the cell nucleus. The Hoechst staining image was first filtered by top-hat and bottom-hat filters to reduce the background, enhanced by contract adjustment, and binarized to quantify the size of nuclei^37^. Cell debris was excluded by their smaller sizes. Live/Dead staining was used to exclude dead cells with dim Live signals and bright dead signals. The cell counting method was modified from our previous works^38–40^. The live cells with nuclei larger than 300 pixels using a 4X objective lens and 1875 pixels using a 10X objective lens (817 µm^2^ area, equivalent to a circle with a diameter of 32 µm) were considered PGCCs, and the others were considered non-PGCCs. The threshold was determined by empirical validation with flow cytometry and visual confirmation (Fig. 1). For time-lapse experiments, Live/Dead staining was not used as that affects cell viability. The nuclear size was determined with the transfected nuclear red fluorescent proteins.

### Flow cytometry and cell sorting

Flow cytometry was performed to quantify and isolate PGCCs and non-PGCCs with and without compound treatments. Becton Dickinson LSR Fortessa II was used for the cellular analysis. Cells were cultured for 24 h, treated with compounds for 48 h, and then stained with 4 μM of Calcein AM, 4 μM of Ethidium homodimer-1 (Invitrogen™, L3224 Live/Dead Viability/Cytotoxicity Kit), and 20 μM of Hoechst 33342 (Thermo Scientific 62249) for 30 min in an incubator. After staining, cells were harvested from culture dishes/plates with 0.05% Trypsin/EDTA (Gibco 25200) and centrifuged at 1000 rpm for 4 min. Then, the cells were re-suspended in PBS (Gibco 10010) supplemented with 1% FBS (Gibco 16000) at a concentration of around 1 × 10^6^ cells per mL. Obtained raw data were analyzed and visualized using FlowJo 10. For the experiments measuring cellular ROS level, 5 μM CellROX™ Deep Red staining reagent (Thermo Scientific C10422) was used. When quantifying PGCCs by flow cytometry, we first examined cellular DNA content by Hoechst staining to identify the G2/M peak (Fig. 1e). The cells having more DNA than those in the G2M phase were considered PGCCs. Dead cells determined by Live/Dead staining were excluded from the analysis. Sony MA900 Cell Sorter was used to isolate PGCCs and non-PGCCs for functional and molecular analysis. To isolate PGCCs and non-PGCCs for single-cell RNA-Seq, stringent cell selection (top 1% Hoechst high for PGCCs and bottom 5% Hoechst low for non-PGCCs) was performed to exclude ambiguous cases. The sorted cells were visually confirmed under the microscope.

### Ploidy evaluation by G-banding metaphase analysis

The cell populations enriched by the PGCCs and by non-PGCCs were obtained after a flow cytometry sorting and placed in 5 mL complete Chang Marrow media (Irvine Scientific 91060). Cells were incubated with 10 µg/mL of ethidium at 37 °C for 90 min and treated with 0.1 µg/mL of Colcemid for 30 min before harvest. Samples were incubated with hypotonic solution (0.075 M KCl) for 25 min and fixed in methanol:acetic acid (3:1). Metaphase cells were spread on a glass slide and stained using a trypsin-Giemsa method. The number of chromosomes in each cell was counted to assess cell ploidy.

### Microfluidic 3D cancer spheroid formation and drug testing on-chip

The 3D cancer spheroids were formed and cultured based on microfluidics we previously developed^41,42^. Following the loading of cancer cells, spheroid formation occurred within microfluidic chambers over the course of one day. Once spheroids were formed, they underwent various treatments, including a DMSO control, a four-day exposure to Docetaxel, and a two-day treatment with anti-PGC compounds, specifically Carfilzomib, ML162, or Thiostrepton, following two days of Docetaxel exposure. After treatment, we stained spheroids with 4 μM of Calcein AM, 4 μM of Ethidium homodimer-1, and 20 μM of Hoechst 33342 for 1 h, followed by fluorescence microscopy imaging. The viability was determined based on the intensity of green fluorescence of Live staining.

### Single-cell RNA-Seq

We utilized flow cytometry to separate PGCCs and non-PGCCs from three breast cancer cell lines: SUM159, MDA-MB-231, and Vari068. Subsequently, we visually confirmed the accurate identification of the flow-sorted PGCCs and non-PGCCs under a microscope. Then, we performed high-throughput single-cell barcoding transcriptome sequencing for each cell population^43–45^. Cells and beads were paired in microwells following our prior works^46,47^, so the mRNA from lysed cells can hybridize onto the barcoded beads. After barcoded beads captured cellular mRNA, we performed RT (Thermofisher Maxima RT kit), PCR (Kapa HiFi Hotstart PCR Readymix), and library preparation (Illumina Nextera XT Library Prep Kit). The cDNA samples were then quantified and pooled by the UPMC Cancer Genome Core for sequencing using the Illumina NextSeq. We obtained approximately 10 million paired-end reads (Read 1: 30 base pairs for the barcode and Read 2: 110 base pairs for mRNA read alignment) for each population. Reads were aligned using STAR with GRCh38.p13 Human reference genome and processed by the standard Dropseq 2.5.1 pipeline. Then, we used the open-source SEURAT 4.0 (http://satijalab.org/seurat/) to analyze single-cell sequencing data^48^. Cells with more than 800 genes detected were considered successfully sequenced, and the cells having more than 5% mitochondrial gene expression were discarded for their poor viability. After a quality check, we got 709 control untreated SUM159 cells, 511 docetaxel-treated SUM159 non-PGCCs, 415 docetaxel-treated SUM159 PGCCs, 549 control untreated MDA-MB-231 cells, 196 docetaxel-treated MDA-MB-231 non-PGCCs, 114 docetaxel-treated MDA-MB-231 PGCCs, 200 control untreated Vari068 non-PGCCs, 126 control untreated Vari068 PGCCs, 291 Docetaxel-treated Vari068 non-PGCCs, and 103 docetaxel-treated Vari068 PGCCs in the experiments.

### mRNA real-time PCR

Total RNA for real-time PCR was extracted and purified using the Purelink RNA Mini Kit (Thermo Fisher). Reverse transcription reactions were performed with M-MLV reverse transcriptase (Life Technology), following the standard protocol using random hexamers (NEB). Real-time PCR was performed with PowerUpTM SYBRTM Green Master Mix labeling in 7500 Fast Real-Time PCR System (Thermo Fisher). PCR conditions were 50 °C for 2 min, 95 °C for 10 min, and 43 cycles of 95 °C for 15 s and 60 °C for 1 min. mRNA expression was normalized against β-actin, allowing comparison of mRNA levels.

### Statistics and reproducibility

Statistical analyses were conducted using R (version 4.1), GraphPad Prism 9, and MATLAB. GraphPad Prism 9 software determined half-maximal inhibitory concentrations (IC50s). Two-tailed Student’s *t*-test compared two groups, while paired 2-way ANOVA and Fisher’s Least Significant Difference (LSD) test compared multiple groups, considering cell line and treatment conditions as variables. Statistical setups varied based on variable types and analysis nature. Within each cell line, treated versus untreated conditions were consistently paired for comparisons, with significance set at *P* < 0.05 (*P < 0.05, **P < 0.01, ***P < 0.001). Standard error of the mean (SEM) represented error bars; sample/group details were specified in Figure Captions. For data with high variability (e.g., gene expression levels), comparisons were made on a log scale. SEURAT in R facilitated single-cell transcriptome sequencing analysis, including outlier detection, hierarchical clustering, principal component analysis (PCA), and Uniform Manifold Approximation and Projection (UMAP). Wilcoxon rank sum test calculated adjusted *P*-values, adjusted via Bonferroni correction using all dataset features. Identifying altered genes involved a logarithmic fold-change of 0.25 and a minimum expression in 10% of cells for pathway analysis. Pathway analysis utilized Enrichr (http://amp.pharm.mssm.edu/Enrichr/) with KEGG 2021 Human and NCI-Nature databases, generating *P*-values via Fisher exact tests.

### Declaration of generative AI in scientific writing

During the preparation of this work, the authors used ChatGPT-3 in order to improve readability and language. After using it, the authors reviewed and edited the content as needed and took full responsibility for the content of the publication.

### Reporting summary

Further information on research design is available in the Nature Portfolio Reporting Summary linked to this article.

## Results

### Quantifying PGCCs and non-PGCCs using a single-cell morphological analysis

To rapidly quantify the number of PGCCs and non-PGCCs, we developed a single-cell morphological analysis pipeline by (1) identifying cell nuclei with Hoechst staining, (2) excluding dead cells with Live/Dead staining, and (3) distinguishing PGCCs and non-PGCCs based on the size of cell nuclei (Fig. 1a). Detailed image processing and representative PGCC, non-PGCC, and dead cell images are presented in Fig. 1, Supplementary Figs. 1 and 2. The threshold to distinguish non-PGCCs and PGCCs was validated using multiple breast cancer cell lines by flow cytometry as well as visual confirmation (Supplementary Fig. 3). Without treatment, SUM159 triple-negative breast cancer (TNBC) cells have less than 0.1% of PGCCs as measured by both single-cell morphological analysis and flow cytometry (Fig. 1b, e and Supplementary Fig. 3). Upon treatment with the conventional chemotherapeutic drug, Docetaxel, there was a decrease in the number of non-PGCCs and an increase in PGCCs. While the increase in PGCCs might be attributed to the selective survival of pre-existing PGCCs, it’s improbable for such expansion to occur within merely 2 days. To substantiate this, time-lapsed single-cell tracking was conducted, confirming the transition from non-PGCCs to PGCCs (Supplementary Movie 1). Based on our observations and existing literature^49^, we posit that the majority of PGCCs following treatment were induced from non-PGCCs. To closely link this research with human breast cancer biology, we included a low-passage TNBC patient-derived cancer cell line (Vari068) in this study. Interestingly, Vari068 carried a large population of PGCCs before treatment, and treatment with Docetaxel further boosted the portion of PGCCs (Fig. 1e and Supplementary Fig. 3). Furthermore, we tested various Hoechst staining concentrations and microscope exposure times to verify that the morphological analysis is insensitive to experimental conditions (Supplementary Fig. 4). Our fluorescence microscope takes 9 min to image a 96-well plate (<6 s per well), including auto-focusing and movement of microscope motorized stage, and an image can be processed within 1 s using our custom MATLAB code. For each experimental condition, we can recognize up to 10,000 cells and quantify the number of PGCCs and non-PGCCs (Fig. 1b). Using the established pipeline, we monitored the development of SUM159 cells with and without Docetaxel treatment (Fig. 1f). Without treatment, SUM159 cells kept growing exponentially and remained non-PGCCs. With Docetaxel treatment, while some cancer cells were killed, a good portion of cells developed into PGCCs. The single-cell morphological analysis pipeline enables high-throughput screening to identify compounds that induce or inhibit PGCC and characterize the dynamics of PGCC development.

### Analyzing compound efficacy comprehensively by counting PGCCs and non-PGCCs

Using the innovative single-cell morphological analysis, we characterized the changes in cell composition when treated with a library of 172 compounds, including conventional chemotherapeutics, ferroptosis inducers, and inhibitors of Mitogen-activated protein kinase (MAPK), Bromodomain and extra-terminal motif (BET), Histone deacetylase (HDAC), Poly (ADP-ribose) polymerase (PARP), Proteasome, Hypoxia-inducible factor (HIF), Aldehyde dehydrogenase (ALDH), Autophagy, Transforming growth factor-beta (TGF-β), CDK4/6, Epidermal growth factor receptor (EGFR), Nuclear factor-kappa B (NF-κB), CXCR1, CXCR4, IL8, Rho-associated protein kinase (ROCK), Colony-stimulating factor-1 receptor (CSF1R), Proto-oncogene tyrosine-protein kinase Src, p21-activated kinase (PAK), Rac, Rho, CDC42, Focal Adhesion Kinase (FAK), Wnt, Aurora, and regulators of key cellular components, including mitochondria, microtubule, integrin, kinesin, and myosin with multiple breast cancer cell lines (Supplementary Data 1). One day after cell loading, cells were treated for two days and then imaged to quantify non-PGCCs and PGCCs (Fig. 2a). Enlarged microscopy images of control and treated cells are illustrated in Fig. 2b, Supplementary Fig. 5. While many compounds killed cancer cells, a significant number of compounds (e.g., Taxanes) induced PGCCs, which could lead to treatment resistance and tumor relapse (Fig. 2c). As reported in literature, Aurora inhibitors (Alisertib and Tozasertib) also significantly boosted the portion of breast cancer PGCCs in our experiments^50^. Among the compounds tested, proteasome inhibitors and ferroptosis inducers effectively inhibited non-PGCCs without inducing PGCCs. Overall, the same trend was observed for 4 TNBC cell types (Fig. 2c, Supplementary Data 2 and 3). In addition to single-dose treatments, 5 concentrations of selected compounds were tested (Fig. 2d and Supplementary Fig. 6). With the increase in concentration, Alisertib killed some non-PGCCs but induced PGCCs. In comparison, Carfilzomib (proteasome inhibitor) and ML162 (ferroptosis inducer) killed both populations as shown by the eradication of the natural PGCC population in Vari068 (Supplementary Fig. 6). We further monitored the dynamics of cell development (Fig. 2e). Under Alisertib treatment, nuclear size kept increasing over 2 days to generate a large population of PGCCs. ML162 killed cells rapidly within 12 h, and Carfilzomib killed cells without inducing PGCCs. The dynamic monitoring provides comprehensive information about the regulation of PGCCs.

### Discovering PGCC inhibitors with screening experiments

As most breast cancer cell lines do not naturally have many PGCCs (<1%), Docetaxel was used to induce PGCCs for discovering anti-PGCC compounds in a library (Fig. 3a and Supplementary Data 1). Enlarged microscopy images of control and treated cells are illustrated in Fig. 3b, Supplementary Fig. 7. As expected, once drug-resistant PGCCs were generated, most therapeutic compounds were no longer effective (Fig. 3c; Supplementary Data 4 and 5). Among the 172 tested compounds, 10 compounds only significantly inhibited PGCCs but not non-PGCCs, and 13 compounds inhibited both PGCCs and non-PGCCs. Previous research has demonstrated that PGCCs are correlated with HIF and EMT, so we tested 11 HIF inhibitors and 5 TGFβ regulators. Unfortunately, only Digoxin, which suppresses the HIF pathway, significantly killed PGCCs. Due to the association between PGCCs and tumor-initiating cells/CSCs, we applied 4 ALDH inhibitors, 2 WNT inhibitors, and 2 CXCR4 inhibitors. While Disulfiram (ALDH inhibitor) could kill PGCCs, all other relevant compounds were ineffective (Fig. 3c; Supplementary Data 4 and 5)^5,21–25^. It was reported that PGCCs have an elevated level of ROS, yet 5 compounds boosting ROS and 5 compounds reducing ROS did not significantly alter PGCC populations at the tested concentration^27,51,52^. Recent studies demonstrated autophagy facilitates cell repair to reduce drug sensitivity, yet 5 tested autophagy inhibitors could not kill PGCCs^53–55^. Among all tested compounds, proteasome inhibitors and ferroptosis inducers are two groups of compounds that were especially effective. All 5 proteasome inhibitors (Bortezomib, MG-132, Carfilzomib, Ixazomib, and Ixazomib Citrate) and 5 ferroptosis inducers (Imidazole ketone erastin (IKE), RSL3, FINO2, ML162, and ML210) significantly inhibited PGCCs (Fig. 3c; Supplementary Data 4 and 5). Additionally, 3 out of 5 HDAC inhibitors (Vorinostat (SAHA), Romidepsin, and Panobinostat) preferably inhibited PGCCs over non-PGCCs. Interestingly, Fulvestrant, which has been used to treat hormone receptor (HR) positive metastatic breast cancer, significantly inhibited PGCCs but not non-PGCCs for TNBC^56^. We also found other regulators of Bcl-2 (ABT-263 and ABT-737), Survivin (YM155), Wee1 (PD0166285 and MK-1775), and NF-κB (Bardoxolone Methyl), and conventional chemotherapeutic drugs, Actinomycin D and SN-38 (active metabolite of irinotecan) significantly killed PGCCs (Supplementary Fig. 7). In addition to single-dose treatment, 5 concentrations of selected compounds were tested to validate the screening experiment (Fig. 3d and Supplementary Fig. 8). We also performed time-lapse monitoring of compound treatment (Fig. 3e). Treatment with Docetaxel killed a good portion of non-PGCCs but not PGCCs. Actinomycin D selectively killed PGCCs but not non-PGCCs, and Carfilzomib and ML162 inhibited both PGCCs and non-PGCCs. The experiments successfully demonstrate our unique capability to distinguish compounds that have different effects on PGCCs and non-PGCCs.

### Validating anti-PGCC compounds

To validate whether the identified compounds only inhibited PGCCs induced by Docetaxel, we tested untreated PGCCs and PGCCs induced by other compounds. In addition to TNBCs, we expanded the study to include HR-positive (T47D and BT474) and human epidermal growth factor receptor 2 (HER2) positive (SKBR3) breast cancer cell lines. As Vari068 naturally has a significant portion of PGCCs, we sorted out untreated PGCCs by flow cytometry and evaluated their treatment responses (Fig. 4a). The untreated PGCCs were more resistant to Docetaxel but responded to Actinomycin D, HDAC inhibitors, proteasome inhibitors, and ferroptosis inducers. The PGCCs induced by other compounds, including chemotherapeutics and aurora inhibitors, were also significantly inhibited by proteasome inhibitors and ferroptosis inducers among 9 breast cancer cell lines (Fig. 4b; Supplementary Figs. 9–14 and Supplementary Tables 2–11). HDAC inhibitors also inhibited PGCCs of TNBC regardless of the induction method (Supplementary Fig. 15 and Supplementary Tables 12–21). In addition to 2D cell culture, we further tested the efficacy of anti-PGCC compounds using a 3D cancer spheroid model (Fig. 4c). Breast cancer cells were loaded into non-adherent microwells to form cancer spheroids. Breast cancer spheroids were treated with DMSO control, 4-day Docetaxel, and 2-day anti-PGCC compounds of Carfilzomib, ML162, or Thiostrepton after 2-day Docetaxel. While Docetaxel treatment killed cancer cells in conventional 2D culture, it was ineffective in inhibiting 3D cancer spheroids. Treatment of anti-PGCC compounds after Docetaxel successfully eradicated treatment-resistant cells induced by Docetaxel. Comprehensive experiments of various methods verified the identified anti-PGCC compounds. More importantly, the identified proteasome inhibitors and ferroptosis inducers are not only effective for TNBCs but also for HR-positive and HER2-positive breast cancer cells.

### Revealing the unique molecular characteristics of PGCCs by scRNA-Seq

To better understand why PGCCs are sensitive to the selected compounds, we performed scRNA-Seq to investigate their unique molecular features. Untreated and Docetaxel-treated PGCCs and non-PGCCs were separated from three breast cancer cell lines (SUM159, MDA-MB-231, and Vari068) using flow cytometry for scRNA-Seq. However, due to the low percentage of PGCCs without treatment (Fig. 1e), insufficient PGCCs were obtained from untreated SUM159 and MDA-MB-231 cell lines, precluding reliable scRNA-Seq analysis for these two cell populations. Microscopic examination confirmed the accurate identification of sorted cells (Supplementary Fig. 16). To further validate the identity of sorted cells, karyotype analysis was performed on the sorted non-PGCCs and PGCCs (Supplementary Fig. 17). The scRNA-Seq results indicate that cell segregation is primarily driven by cell lines and treatment conditions, given the inherent similarities between PGCC and non-PGCC sub-populations within a cell line. However, PGCCs and non-PGCCs of the same cell line are still segregated (Fig. 5a and Supplementary Fig. 18). We identified the marker genes that distinguish PGCCs from non-PGCCs and performed pathway analysis using individual cell lines as well as the overlapping markers between three cell lines (Fig. 5b, c and Supplementary Tables 22–25). Using the KEGG 2021 Human Pathway database, the pathways of “Cell cycle,” “Oxidative phosphorylation,” “Ribosome,” and “Ferroptosis” were highlighted. Using the NCI-Nature pathway database, the pathways of “PLK1 signaling,” “FOXM1 transcription factor,” “Aurora signaling,” and “HDAC signaling” were highlighted. We also specifically identified the top-ranked altered genes related to polyploidy and aneuploidy (polo-like kinase 1 (PLK1), cell division cycle protein 20 (CDC20), and Cyclin B1 (CCNB1)), cell cycle and anaphase-promoting complex (APC) (Cyclin Dependent Kinase Inhibitor 3 (CDKN3), Ubiquitin Conjugating Enzyme E2C (UBE2C), and Pituitary tumor transforming gene 1 (PTTG1)), ferroptosis (Ferritin Light Chain (FTL), Ferritin Heavy Chain 1 (FTH1), and Solute Carrier Family 3 Member 2 (SLC3A2)), and ROS generation (Reactive Oxygen Species Modulator 1 (ROMO1)) (Fig. 5d). The expression levels of other genes relevant to EMT, CSC, cell cycle, metabolism, ribosome, and ferroptosis are plotted in Supplementary Figs. 19–22. We validated 3 genes (CCNB1, CDC20, and FTH1) with qRT-PCR, and the altered gene expression matched well with the scRNA-Seq data (Supplementary Fig. 23 and Supplementary Table 26). Based on the transcriptome analysis, we tested a FOXM1 inhibitor, Thiostrepton. When treating cells without Docetaxel pre-treatment, it killed non-PGCCs without inducing PGCCs. When treating cells pre-treated with Docetaxel, it selectively inhibited PGCCs but not non-PGCCs (Fig. 5e). The scRNA-Seq experiments successfully revealed the unique molecular profile of PGCCs for guiding therapeutic strategies.

### The elevated ROS level of PGCCs causes vulnerability to ferroptosis

Based on the transcriptome analysis and literature^57^, we hypothesized the altered metabolism and ferroptosis regulators of PGCCs are the cause of ferroptosis sensitivity. To test this hypothesis, we first incorporated a deep red cellular ROS measurement dye in the single-cell morphological analysis (Fig. 6a). In this 4-color experiment, the ROS level of each cell can be quantified, and PGCCs were found to have a significantly higher ROS level with and without the treatment of Docetaxel (Fig. 6b). The finding was validated with flow cytometry (Supplementary Fig. 24). The elevated ROS can induce lipid peroxidation and sensitize ferroptosis, which is a promising strategy to inhibit PGCCs^58^. To further test the relationship between ROS and ferroptosis, we applied Rotenone and GSK2795039 to boost and reduce ROS respectively (Supplementary Fig. 25). Without Docetaxel treatment, the ROS reduction successfully rescued cells from ferroptosis, and the boost further sensitized cells to ferroptosis (Fig. 6c, Supplementary Fig. 26 and Supplementary Table 27). We also tested the effects on PGCCs induced by Docetaxel and Alisertib, and ROS reduction still rescued cells from ferroptosis (Fig. 6d; Supplementary Fig. 26 and Supplementary Tables 28 and 29). However, as the cellular ROS level was already boosted by Docetaxel, Rotenone could not further boost ROS to sensitize ferroptosis. The relationship holds true for 4 cell lines among TNBCs, HR-positive, and HER2-positive breast cancer. The preliminary mechanistic investigation highlights the unique metabolic characteristics of PGCCs and ferroptosis as a strategy to kill treatment-resistant PGCCs utilizing this feature.

## Discussion

While the importance of PGCCs in cancer treatment resistance has been widely acknowledged, there is no effective way to eradicate them. The investigation is hindered by the lack of an effective screening method to quantify PGCCs. Although conventional drug screening assays (e.g., MTT, XTT, or ATP) rapidly measure the overall inhibition of cancer cells, the assays are unable to tell whether a small number of PGCCs were eradicated. The compounds selected in this manner might leave or induce treatment-resistant PGCCs, eventually causing relapse. As such, the missing single-cell resolution can be detrimental to cancer drug discovery. Currently, flow cytometry is the gold standard in quantifying PGCCs by their elevated fluorescent intensity of Hoechst/DAPI. While flow cytometry offers the theoretical capability to analyze tens of thousands of cells per second, practical constraints arise when transitioning (washing and cleaning steps) between multiple samples, particularly when conducting high-throughput compound screening. High-throughput flow cytometry machines like the ZE5 Cell Analyzer take a minimum of 15 min to characterize a plate. In contrast, our single-cell morphological method excels in speed, requiring less than 6 s to image a condition and less than 1 s to analyze the image. A single image can recognize and classify up to 10,000 cells as PGCCs, non-PGCCs, or dead cells. As it stands, our method is already significantly faster than flow cytometry. More importantly, advancements in microscopy imaging technology and the computational capabilities of integrated circuits (ICs) continue to progress. Historically, IC power has doubled approximately every 18 to 24 months. This trend indicates that scientific cameras will offer even greater throughput and improved imaging quality in the future. Concurrently, improvements in computational resources and algorithms enhance the pace of cellular image analysis. In contrast, flow cytometry throughput remains constrained by the physical washing and cleaning steps involved. Additionally, flow cytometry relies on the absolute fluorescent intensity of DAPI/Hoechst for PGCC determination, which can be significantly impacted by staining conditions, necessitating meticulous calibration with controls. Single-cell morphological analysis is capable of measuring both nuclear morphology and DAPI/Hoechst intensity, and these metrics are less sensitive to variations in fluorescence staining and exposure protocols (Supplementary Fig. 4). While our current focus centers on nuclear size in this study, the data we have gathered on nuclear morphology opens the door to more comprehensive PGCC analysis, such as distinguishing PGCCs with a single giant nucleus from those with multiple nuclei. Furthermore, our method enables successful time-lapse experiments to monitor the dynamic transition of cell populations, a task not easily accomplished with flow cytometry. In summary, our developed method offers rapid and reliable quantification of PGCCs, surpassing existing techniques and expediting the discovery of PGCC inhibitors.

In addition to single-cell morphological analysis, we performed scRNA-Seq to characterize individual breast PGCCs and non-PGCCs. Although we stringently selected PGCCs (top 1% Hoechst high) and non-PGCCs (bottom 5% Hoechst low), some non-PGCCs still have similar molecular profiles as PGCCs. The observation supports that some small non-PGCCs might be generated through budding from large PGCCs^20^. The significant cellular heterogeneity of PGCCs and non-PGCCs could not be revealed using conventional bulk sequencing. While it was reported that PGCCs are associated with EMT and CSC, the relevant pathways and genes were not highly ranked to distinguish PGCCs with Docetaxel treatment (Supplementary Figs. 19–22). The lack of transcriptome alteration in EMT and CSC pathways matches our functional tests which conclude that the inhibitors of HIF, TGFβ, ALDH, WNT, and CXCR4 could not significantly inhibit breast PGCCs. Interestingly, the untreated Vari068 PGCCs showed up-regulation of Vimentin and CD44 and down-regulation of EPCAM and KRT19, which are correlated with EMT, yet the EMT-relevant pathways are still not top-ranked (Supplementary Fig. 21 and Supplementary Tables 22–25). Given the variation between cell lines, the pathways of “Cell cycle,” “Oxidative phosphorylation,” “Ribosome,” and “Ferroptosis” in the KEGG 2021 and “PLK1 signaling,” “FOXM1 transcription factor,” “Aurora signaling,” and “HDAC signaling” in the NCI-Nature pathway databases are central to the difference between PGCCs and non-PGCCs. Based on the pathway analysis, we successfully identified a selective PGCC inhibitor, Thiostrepton, through inhibition of FOXM1 (Fig. 5e). In addition, PLK1 was unanimously up-regulated in PGCCs among all cell lines. PLK1 overexpression is known to increase the size and/or number of centrosomes, which causes aneuploidy/polyploidy and tumorigenesis through improper segregation of chromosomes^59^. In our data, the expression levels of various centromeres were also altered in PGCCs, supporting the role of centrosomes in generating and maintaining PGCCs. In addition, PLK1 was up-regulated by CoCl2 treatment, which generated PGCCs, and PLK1 blockage sensitized glioblastoma cells to ionizing radiation^60,61^. This evidence supports the critical role of PLK1 in regulating PGCCs. Based on this observation, we tested two PLK1 inhibitors, BI2536 and BI6727, but they did not significantly inhibit PGCCs (Supplementary Data 4 and 5). Further exploration might be performed in this direction. In addition to PLK1, PGCCs up-regulated CDC20 and CCNB1, which are associated with aneuploidy/polyploidy, adverse clinical outcomes of breast cancer patients, and resistance to adjuvant therapy^62–64^. PGCCs also up-regulated genes associated with APC, including CDKN3, UBE2C, and PTTG1. The altered cell cycle regulators highlight potential strategies to inhibit PGCCs. PGCCs also transformed metabolism in ribosomes and oxidative phosphorylation. The overexpression of large ribosomal subunits in breast cancer CTCs was reported to enhance metastatic growth^65^. The transformed oxidative phosphorylation contributes to high ROS levels in PGCCs. For example, high ROMO1 induces ROS production in mitochondria^66^. Interestingly, PGCCs also showed low expression of ferroptosis regulators (FTL, FTH1, and SLC3A2), which is a feature of ferroptosis-sensitive cells^67^. This observation matches with the vulnerability of PGCCs to ferroptosis inducers in the functional tests. Overall, the scRNA-Seq experiment revealed the unique characteristics of PGCCs. While not all targets are immediately druggable, the observations open avenues for therapeutic strategies.

Based on the functional screening and transcriptome analysis, we identified 3 classes of compounds that effectively killed PGCCs: HDAC inhibitors, proteasome inhibitors, and ferroptosis inducers. Histone deacetylation is an important regulator of gene expression, and overexpression of HDACs was observed in various types of solid tumors, including breast cancer^68^. HDAC inhibitors are known to inhibit the proliferation of tumor cells and suppress the self-renewal and expansion of CSCs and EMT that might drive cancer invasion and metastasis^69–71^. It was reported that HDAC inhibitors induce polyploidy in breast cancer cells^72^. However, in our experiments, HDAC inhibitors significantly inhibited Docetaxel-induced PGCCs and also flow-sorted Vari068 PGCCs. The difference might be caused by different methods to quantify PGCCs, and the prior study focused on p21−/− or p53−/− cell populations. In clinical trials, the efficacy of single-agent HDAC inhibitors is limited by the lack of response and development of treatment resistance, yet the combination of HDAC inhibitors with chemotherapy and radiotherapy has demonstrated promising results in both preclinical and clinical studies^73^. The combination of HDAC inhibitors with exemestane and paclitaxel shows clinical benefits and tolerable side effects in clinical trials of breast cancer^74,75^. While the PGCCs were not investigated in the trials, the delayed tumor relapse and clinical benefits might be attributed to the eradication of PGCCs by HDAC inhibitors.

In addition to HDAC inhibitors, proteasome inhibitors significantly killed PGCCs for all 9 cell lines we tested. While polyploidy was reported to be associated with Bortezomib resistance in a myeloma cell line, our observations in breast cancer cell lines are different^76^. Proteasomes, which are responsible for the degradation of proteins, are essential for the maintenance of cellular homeostasis^77^. Inhibition of proteasome machinery can break the subtle balance and induce malignant cells towards cell death. In preclinical models, cancer cells were found to be more susceptible to proteasome inhibitors than non-malignant cells, so proteasome inhibition holds promise to treat cancer^78^. While proteasome inhibition alters the degradation of many proteins, it was highlighted that proteasome inhibition benefits cancer patients by repressing NF-κB signaling, which correlates with cancer proliferation, metastasis, and avoidance of apoptosis^79^. In this study, we tested 4 NF-κB regulators (Bardoxolone Methyl, TPCA-1, QNZ, and Betulinic acid), yet only Bardoxolone Methyl significantly inhibited PGCCs. Transcriptome analysis also did not find NF-κB pathways altered in PGCCs. Overall, our preliminary data do not support PGCC inhibition by proteasome inhibitors through NF-κB. Proteasome inhibition was also reported to accumulate pro-apoptotic Bcl-2-associated X (Bax) protein, which inhibits anti-apoptotic Bcl-2 protein to activate the caspase cascade for apoptosis^80^. Three tested Bcl-2 inhibitors all inhibited PGCCs in our study, and the effects of two (ABT-263 and ABT-737) were significant. The scRNA-Seq experiment also highlighted the cell cycle pathway, which heavily interacts with Bcl-2^81^. These preliminary results support the role of Bax and Bcl-2 in killing PGCCs by proteasome inhibition. Given the preclinical evidence, clinical trials were conducted on various malignancies, and Bortezomib was approved by the US FDA to treat multiple myeloma. However, treating solid tumors with proteasome inhibitors alone has had limited success so far. In breast cancer, Bortezomib was tested as a single agent as well as in combination with pegylated liposomal doxorubicin. Both clinical trials showed the treatment was well tolerated yet provided limited clinical activity against metastatic breast cancer^82,83^. The limited clinical success was caused by the unfavorable distribution of bortezomib in solid tumors, which might be resolved by a more precise drug delivery mechanism and/or an elevated dose of the second-generation proteasome inhibitors (e.g., Carfilzomib or Ixazomib), which are less toxic^77^. Recently, Bortezomib was found to enhance the effectiveness of fulvestrant to treat aromatase-inhibitor-resistant, ER-positive metastatic breast cancer^84^. The clinical trials suggest that proteasome inhibitors are not good first-line drugs for all breast cancer patients, yet they can be effective for treatment-resistant patients. The idea of treating resistant diseases matches our observation that proteasome inhibitors are effective in killing treatment-resistant PGCCs. It would be promising to test the efficacy of proteasome inhibitors for resistant patients with a large population of PGCCs in future clinical trials.

Ferroptosis inducer is the other class of compounds that effectively eradicated PGCCs in our study. Ferroptosis is iron-dependent non-apoptotic cell death and features excess lipid peroxidation and elevated ROS^85,86^. In our studies, PGCCs were found to under-express ferroptosis regulators (FTL, FTH1, and SLC3A2) and up-regulate the ROS level, which are characteristics of ferroptosis-sensitive cells^67^. In addition, while PGCCs could survive Docetaxel treatment, their ROS level was further up-regulated (Fig. 6b). The observation matches well with literature^57^. This metabolic characteristic makes treatment-resistant PGCCs vulnerable to ferroptosis. We tested 5 ferroptosis inducers based on 2 different mechanisms: inhibition of Glutathione Peroxidase 4 (RSL3, ML162, FINO2, and ML210) and System Xc- (IKE)^86,87^. All 5 compounds significantly killed breast PGCCs. While it was reported that ferroptosis inducers kill breast cancer cells, its efficacy on PGCCs was reported in this work^88,89^. The capability to kill PGCCs highlights the clinical potential of ferroptosis inducers to treat resistant diseases. So far, there is no clinical trial demonstrating the value of ferroptosis-associated agents to enhance patient outcomes^67,87^. Although the application of ferroptosis agents is currently restricted by limited water solubility of agents and toxicity on kidneys, neutralizers to avoid excessive ferroptosis and novel inducers and drug delivery methods can potentially alleviate these concerns^90,91^.

In summary, we have developed a single-cell morphological analysis pipeline for the swift differentiation of compounds targeting non-PGCCs, PGCCs, or both. This method allowed us to explore numerous potential anti-PGCC strategies, unveiling potent compounds to combat cancer treatment resistance. We acknowledge the lack of animal experiments is a limitation, yet we aim to promptly share our method, screening outcomes, and scRNA-Seq results for the greater benefit of the PGCC research community. Further mechanistic and in vivo investigations will be performed in our future research endeavors. Overall, this workflow holds significant applicability in diverse high-throughput screening experiments for quantifying varied cellular populations and their responses to treatments.

### Supplementary information

Supplementary Information

Description of Additional Supplementary Files

Supplementary Movie 1

Supplementary Data 1

Supplementary Data 2

Supplementary Data 3

Supplementary Data 4

Supplementary Data 5

Supplementary Data 6

Supplementary Software 1

Reporting Summary

## Data Availability

The authors declare that all relevant data are included in the main text and/or its supplementary information files. Source data will be available in Supplementary Data 6. The single-cell RNA-Seq data have been uploaded to the gene expression omnibus Series GSE248717.

## Code availability

The custom MATLAB code is provided as Supplementary Software 1.
