# Supplementary material for: Single-cell morphological and transcriptome analysis unveil inhibitors of polyploid giant breast cancer cells in vitro
Source: Commun Biol. 2023 Dec 21;6:1301. doi: 10.1038/s42003-023-05674-5 (PMC10739852; doi:10.1038/s42003-023-05674-5)
Supplement: Supplementary file 1 — Supplementary Information [file 42003_2023_5674_MOESM1_ESM.pdf]

Title:

Single-Cell Morphological and Transcriptome Analysis Unveil Inhibitors of Polyploid Giant Breast Cancer Cells In Vitro

Authors:

Mengli Zhou[1,2,3], Yushu Ma[1,2], Chun-Cheng Chiang[1,2], Edwin C. Rock[4], Samuel Charles Butler[1], Rajiv Anne[4], Svetlana Yatsenko[5], Yinan Gong[1,6], Yu-Chih Chen[1,2,4,7*]

1 UPMC Hillman Cancer Center, University of Pittsburgh, 5115 Centre Ave, Pittsburgh, PA 15232, USA;

2 Department of Computational and Systems Biology, University of Pittsburgh, 3420 Forbes Avenue, Pittsburgh, PA 15260, USA;

3 Xiangya Hospital, Central South University, Changsha, Hunan, 410008, China;

4 Department of Bioengineering, Swanson School of Engineering, University of Pittsburgh, 3700 O'Hara Street, Pittsburgh, PA 15260, USA;

5 Department of Pathology, University of Pittsburgh, Pittsburgh, PA, USA; Department of Obstetrics, Gynecology and Reproductive Sciences, University of Pittsburgh, Pittsburgh, PA, USA; Magee Womens Research Institute, Pittsburgh, PA, USA;

6 Department of Immunology, University of Pittsburgh School of Medicine, Pittsburgh, PA 15261, USA;

7 CMU-Pitt Ph.D. Program in Computational Biology, University of Pittsburgh, 3420 Forbes Avenue, Pittsburgh, PA 15260, USA.

*Corresponding author

Yu-Chih Chen

5115 Centre Ave, Pittsburgh, PA 15232, USA

Tel: 412-623-7701; E-mail: cheny25@upmc.edu.

Supplementary Information

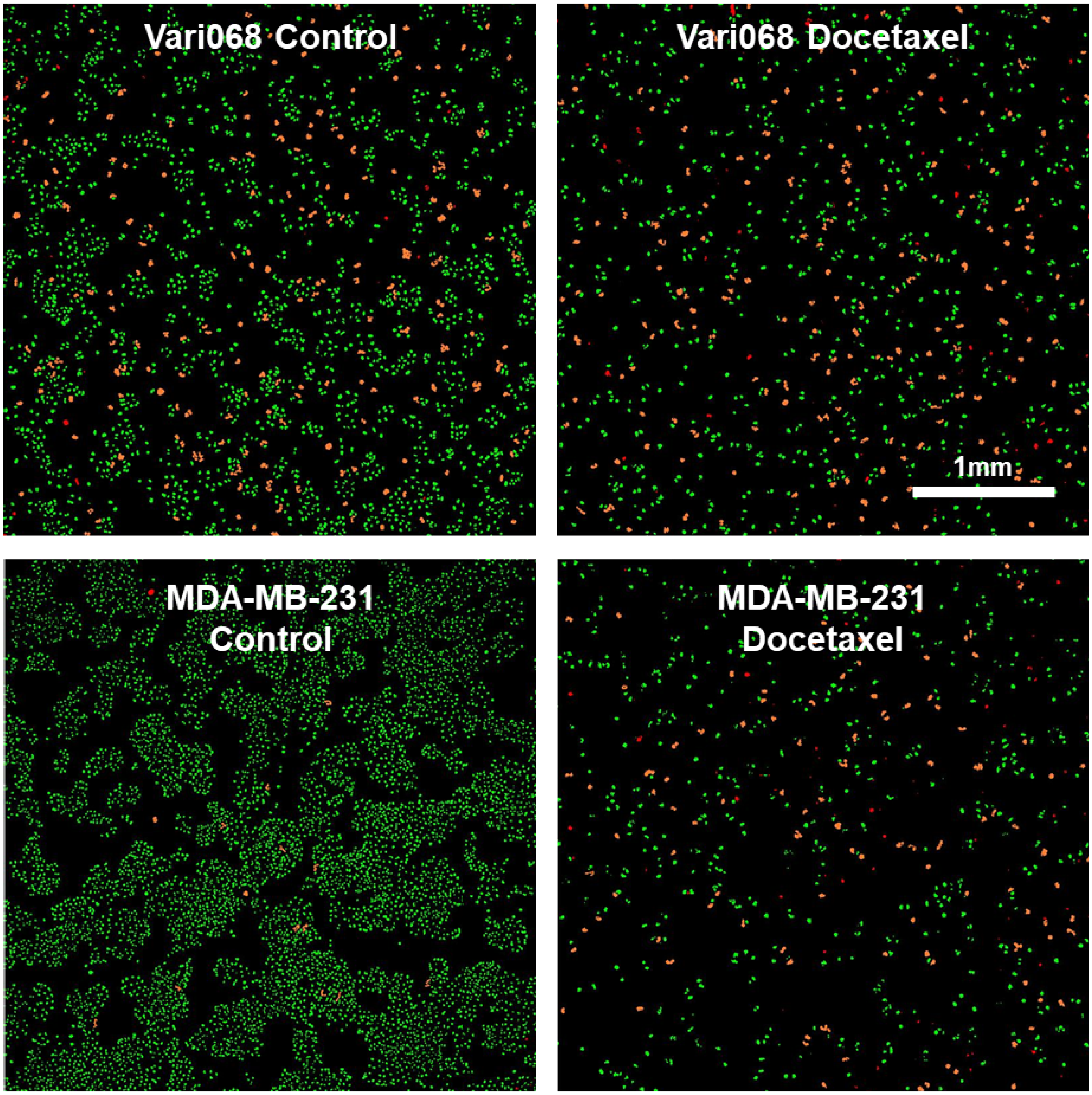

Supplementary Figure 1 Representative Images of Vari068 control and with Docetaxel treatment; MDA-MB-231 control and with Docetaxel treatment. For Vari068, without treatment, 2,904 non-PGCCs, 186 PGCCs, and 15 dead cells were recognized. With Docetaxel treatment, the population was shifted to 905 non-PGCCs, 155 PGCCs, and 124 dead cells. For MDA-MB-231, without treatment, 5,108 non-PGCCs, 4 PGCCs, and 18 dead cells were recognized. With Docetaxel treatment, the population was shifted to 478 non-PGCCs, 69 PGCCs, and 106 dead cells. Green dots represent non-PGCCs, orange dots represent PGCCs, and red dots represent dead cells (Scale bar: 1 mm)

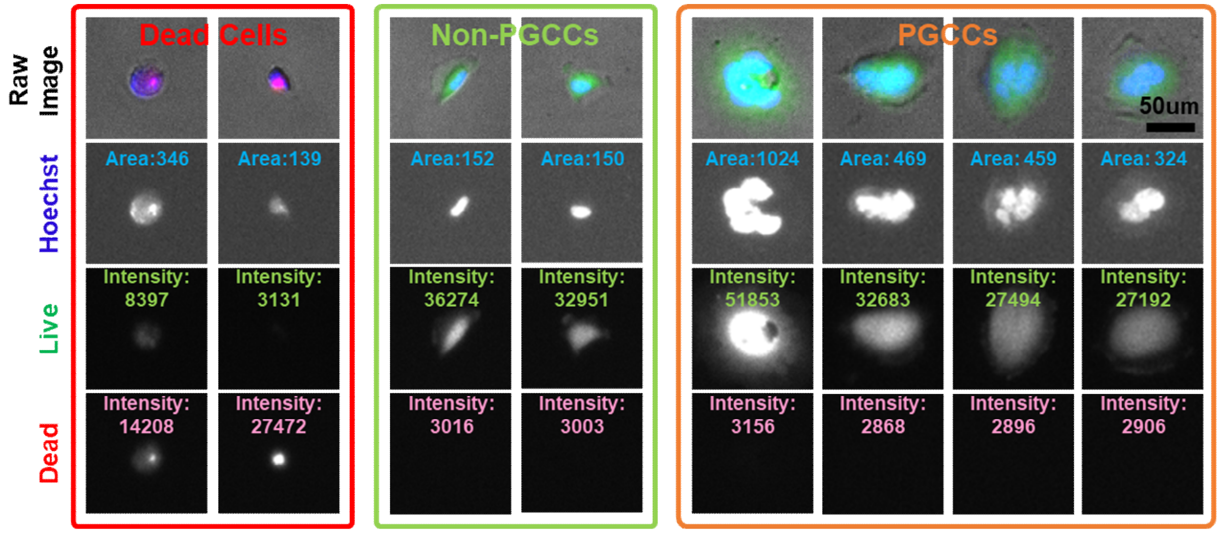

Supplementary Figure 2 The detailed image processing and representative PGCC, non-PGCC, and dead cell images. (Scale bar: 50 μm)

a

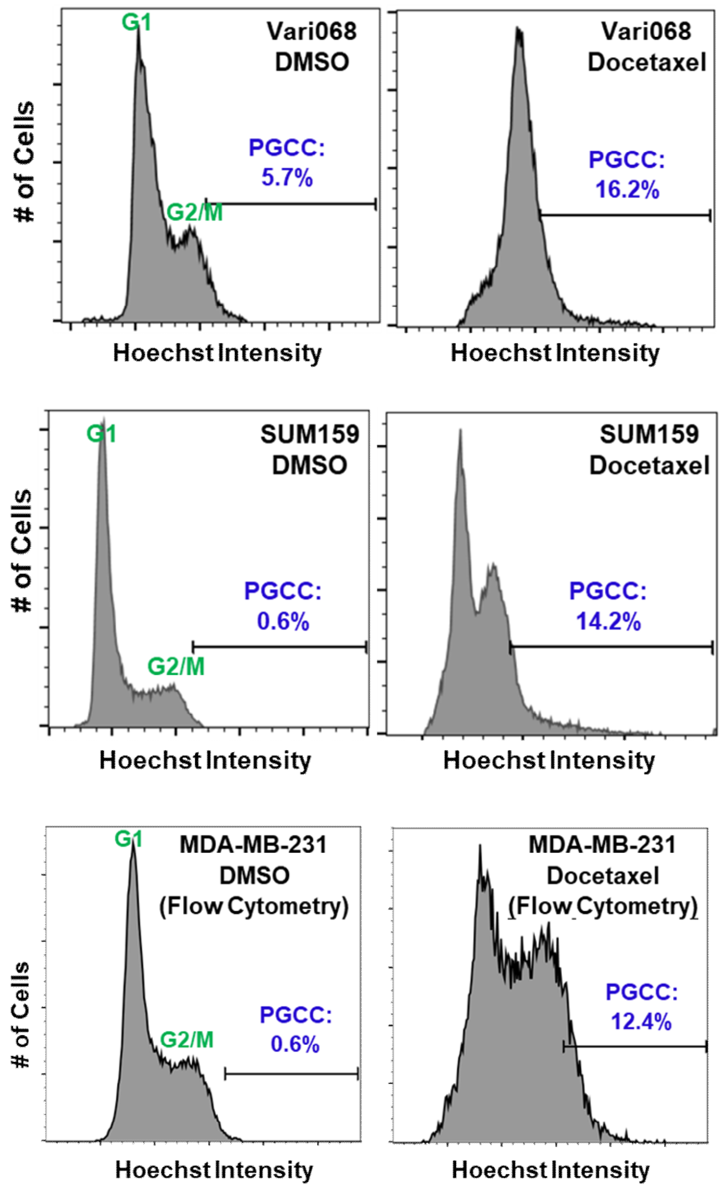

Vari068
DMSO
G1
G2/M
PGCC:
5.7%
# of Cells
Hoechst Intensity
Vari068
Docetaxel
PGCC:
16.2%
Hoechst Intensity
SUM159
DMSO
G1
G2/M
PGCC:
0.6%
# of Cells
Hoechst Intensity
SUM159
Docetaxel
PGCC:
14.2%
Hoechst Intensity
MDA-MB-231
DMSO
(Flow Cytometry)
G1
G2/M
PGCC:
0.6%
# of Cells
Hoechst Intensity
MDA-MB-231
Docetaxel
(Flow Cytometry)
PGCC:
12.4%
Hoechst Intensity

b

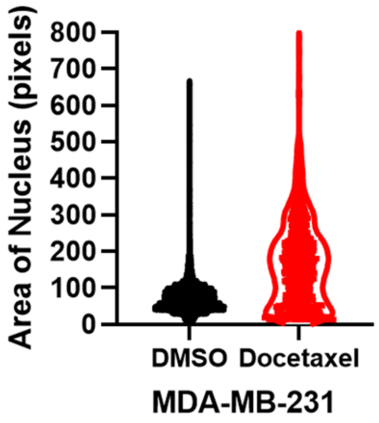

Area of Nucleus (pixels)
800
700
600
500
400
300
200
100
0
DMSO
Docetaxel
MDA-MB-231

Supplementary Figure 3. Validation experiments of single-cell morphological analysis and flow cytometry. (a) Flow cytometry for MDA-MB-231. Identification of PGCCs by flow cytometry according to the fluorescent intensity of Hoechst staining. The live cells having stronger Hoechst signal than those in the G2/M phase were considered PGCCs. X-axis represents the intensity of Hoechst (DAPI blue), and Y-axis represents cell numbers. (b) Identification of PGCCs and non-PGCCs by single-cell morphological analysis according to the area of cell nuclei. MDA-MB-231 breast cancer cells were treated with DMSO control (n = 5,970 cells) and 1 μM of Docetaxel (n = 549 cells).

a

b

c

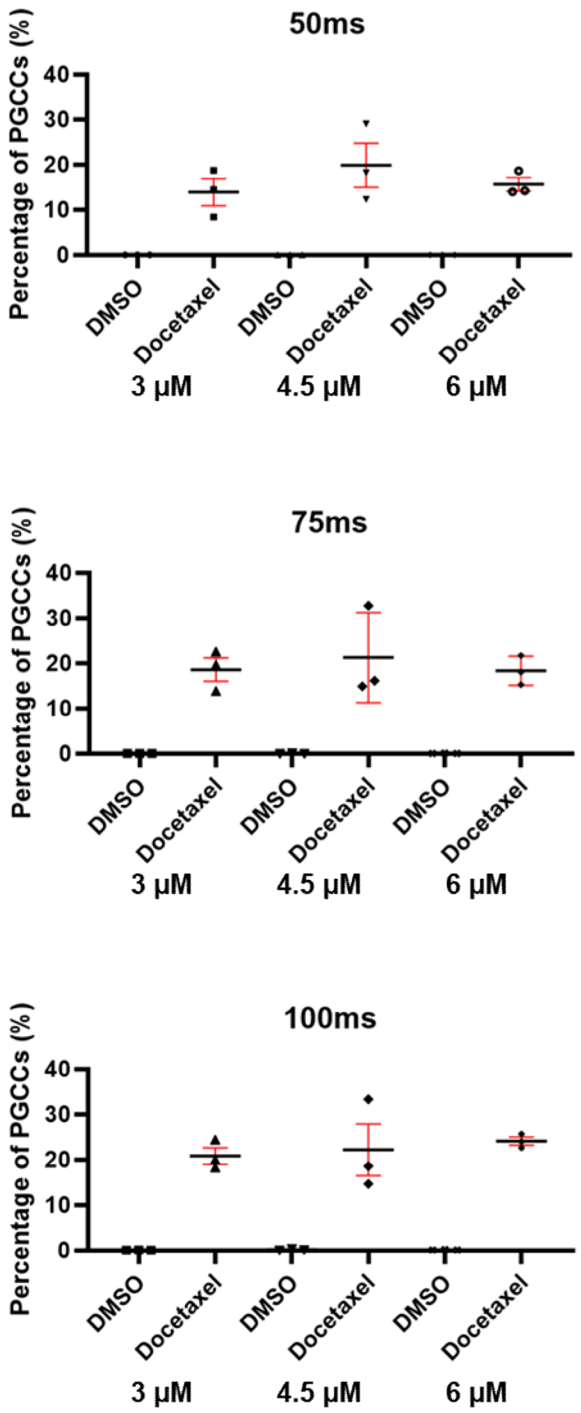

Supplementary Figure 4. Validation experiments of morphological analysis. a. The microscopy exposure time is 50 ms b. The microscopy exposure time is 75 ms c. The microscopy exposure time is 100 ms. X-axis represents different Hoechst staining concentrations (3 μM, 4.5 μM, 6 μM) and different treatments (DMSO control, Docetaxel). Y-axis represents the percentage of PGCCs (%). Error bars indicate standard error of the mean (SEM). N = 3.

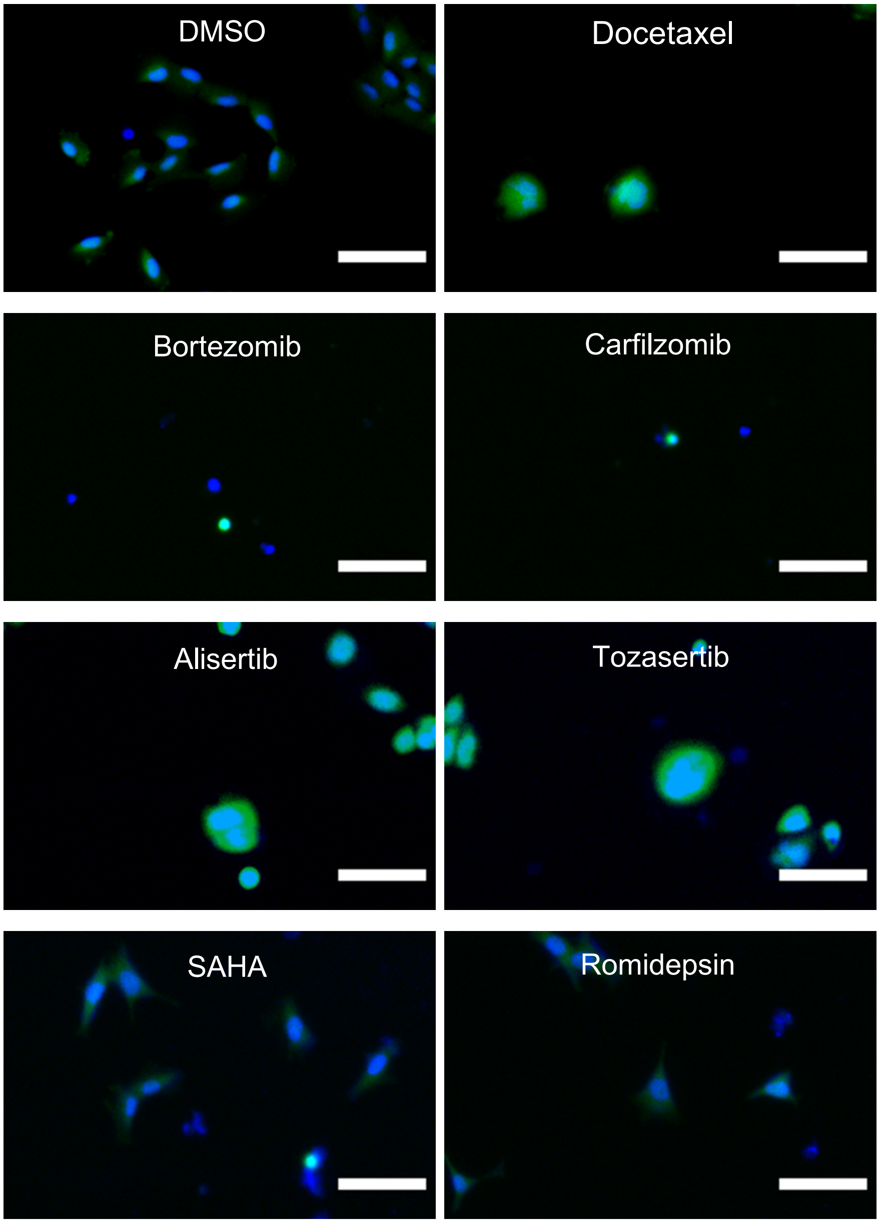

Supplementary Figure 5. Representative Images of Compounds cytotoxic effects screening Experiments Representative images of SUM159 treated with DMSO control, 1 µM of Docetaxel, Bortezomib and Carfilzomib (proteasome inhibitors), Alisertib and Tozasertib (aurora inhibitors), SAHA and Romidepsin (HDAC inhibitors). Cells were stained with LIVE (green), Dead (red) and Hoechst (blue) staining. (Scale bar: 100 µm)

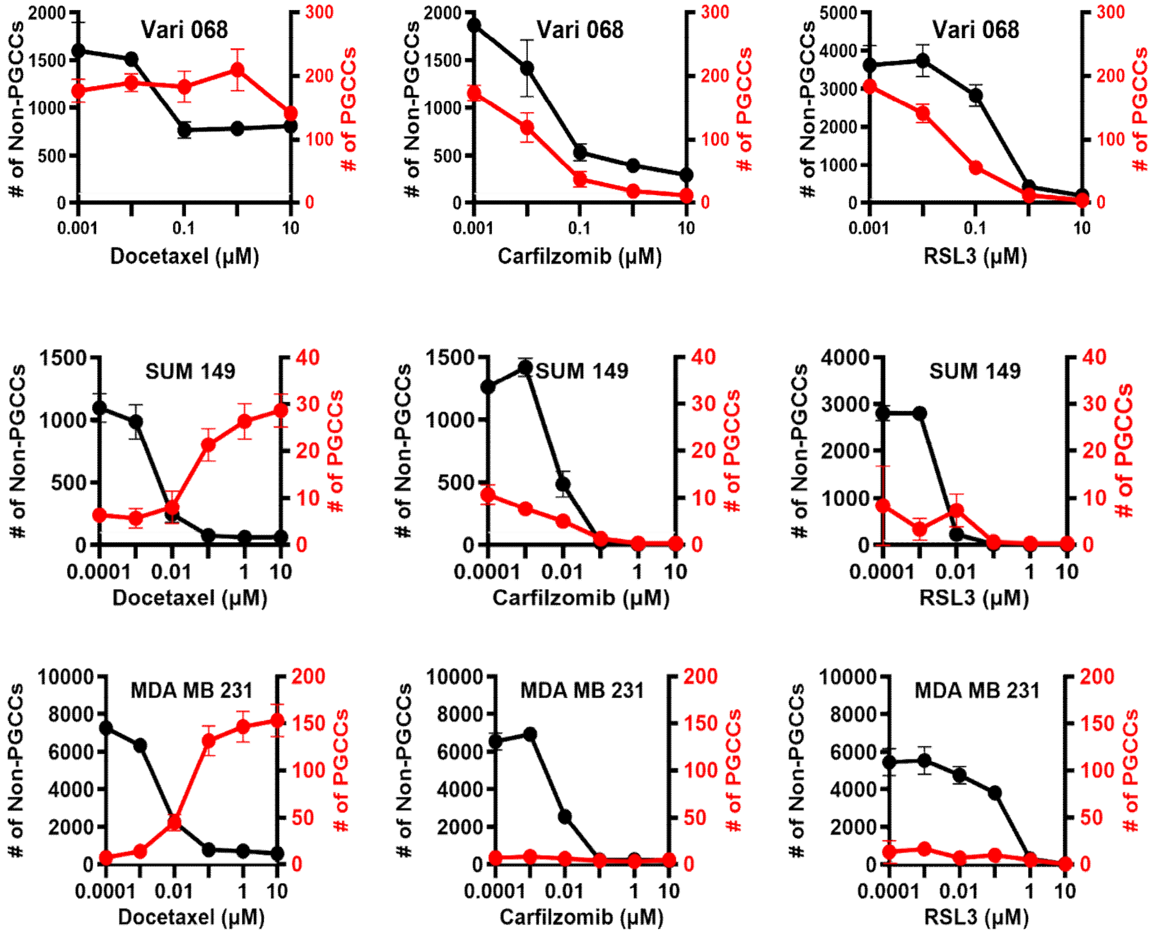

Supplementary Figure 6. Cytotoxicity tests using Vari068, SUM149, and MDA-MB-231. Cytotoxicity tests of selected compounds with 6 concentrations using Vari 068, SUM149 and MDA-MB-231 cells. Docetaxel, Carfilzomib, and RSL3 were selected to test 6 concentrations: 10 μM, 1 μM, 0.1 μM, 0,01 μM, 0.001 μM and 0.0001 μM. The black curve is plotted based on the number of non-PGCCs, and red curve is plotted based on the number of PGCCs. The X-axis represents concentrations of the selected compounds. The left Y-axis (black) represents the number of non-PGCCs, and the right Y-axis (red) represents the number of PGCCs. Error bars indicate the standard error of the mean (SEM). N = 3.

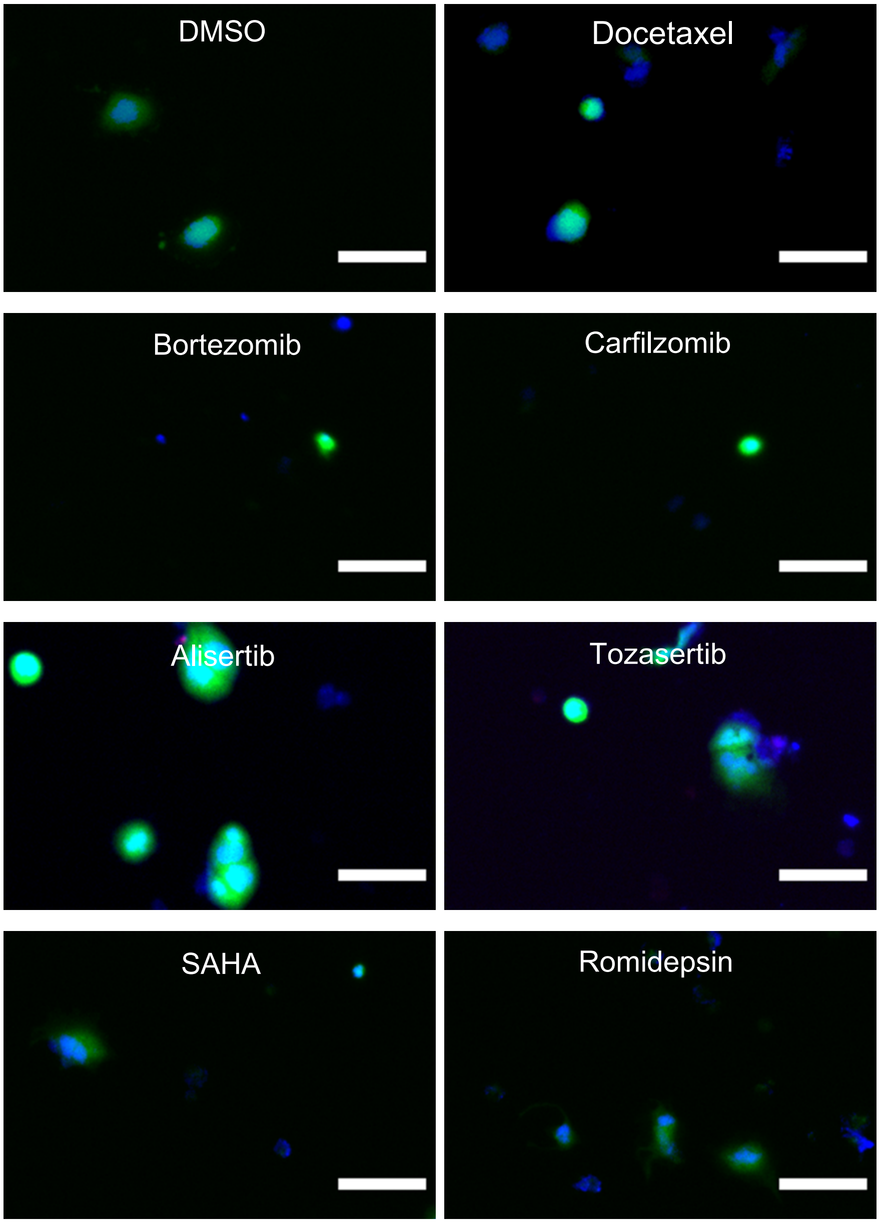

Supplementary Figure 7. Representative Images of Discover Screening for Anti-PGCCs Compounds. Representative images of SUM159 treated with DMSO control, 1 μM of Docetaxel, Bortezomib and Carfilzomib (proteasome inhibitors), Alisertib and Tozasertib (aurora inhibitors), SAHA and Romidepsin (HDAC inhibitors). Cells were stained with LIVE (green), Dead (red) and Hoechst (blue) staining. (Scale bar: 100 μm)

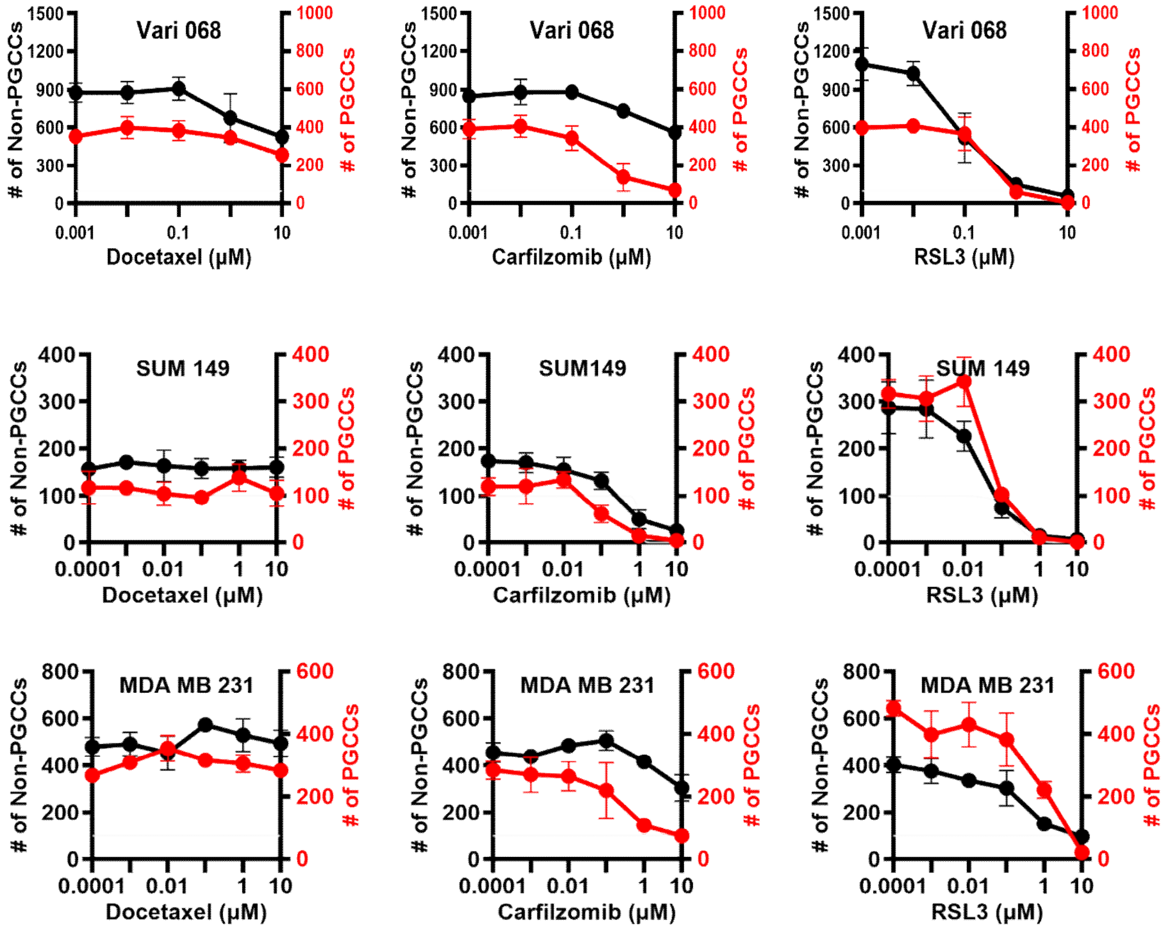

Supplementary Figure 8. Inhibition effects of PGCCs tests using Vari068, SUM149 and MDA-MB-231. Inhibition of PGCCs tests of selected compounds with 6 concentrations using Vari068, SUM149 and MDA-MB-231 cells. Docetaxel, Carfilzomib, and RSL3 were selected to test 6 concentrations: 10 μM, 1 μM, 0.1 μM, 0,01 μM, 0.001 μM and 0.0001 μM. Black curve is plotted based on the number of non-PGCCs, and red curve is plotted based on the number of PGCCs. X-axis represents concentrations of the selected compounds. Left Y-axis (black) represents the number of non-PGCCs, and right Y-axis (red) represents the number of PGCCs. Error bars indicate standard error of the mean (SEM). N = 3.

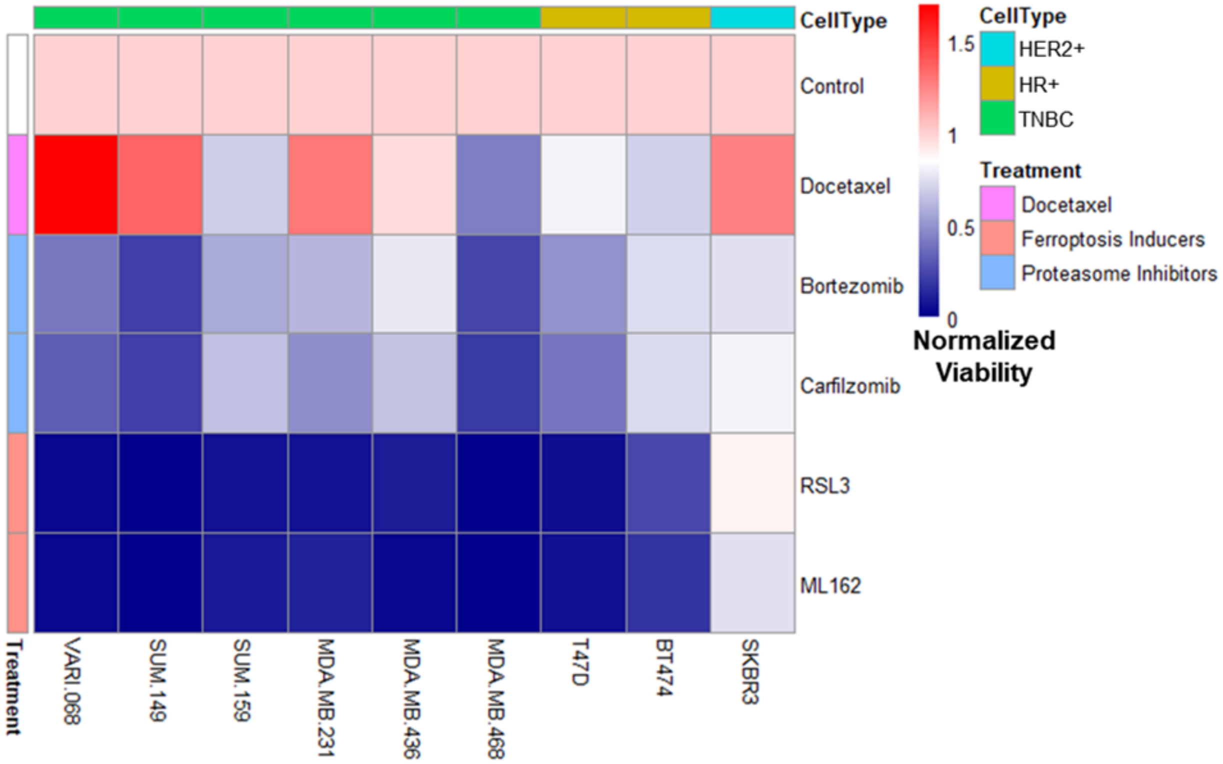

Supplementary Figure 9. Validation of anti-PGCC compounds for non-PGCCs with Docetaxel treatment. Nine breast cancer cell lines (hormone receptor (HR) positive (T47D and BT474), HER2 positive (SKBR3), and TNBC (Vari068, SUM149, SUM159, MDA-MB-231, MDA-MB-436, and MDA-MB-468) were tested. Color gradient represents the cell viability normalized to the control. Different cell lines treated with Docetaxel were resistant to Docetaxel yet responded to proteasome inhibitors and ferroptosis inducers. Statistics is provided in Supplementary Table 7.

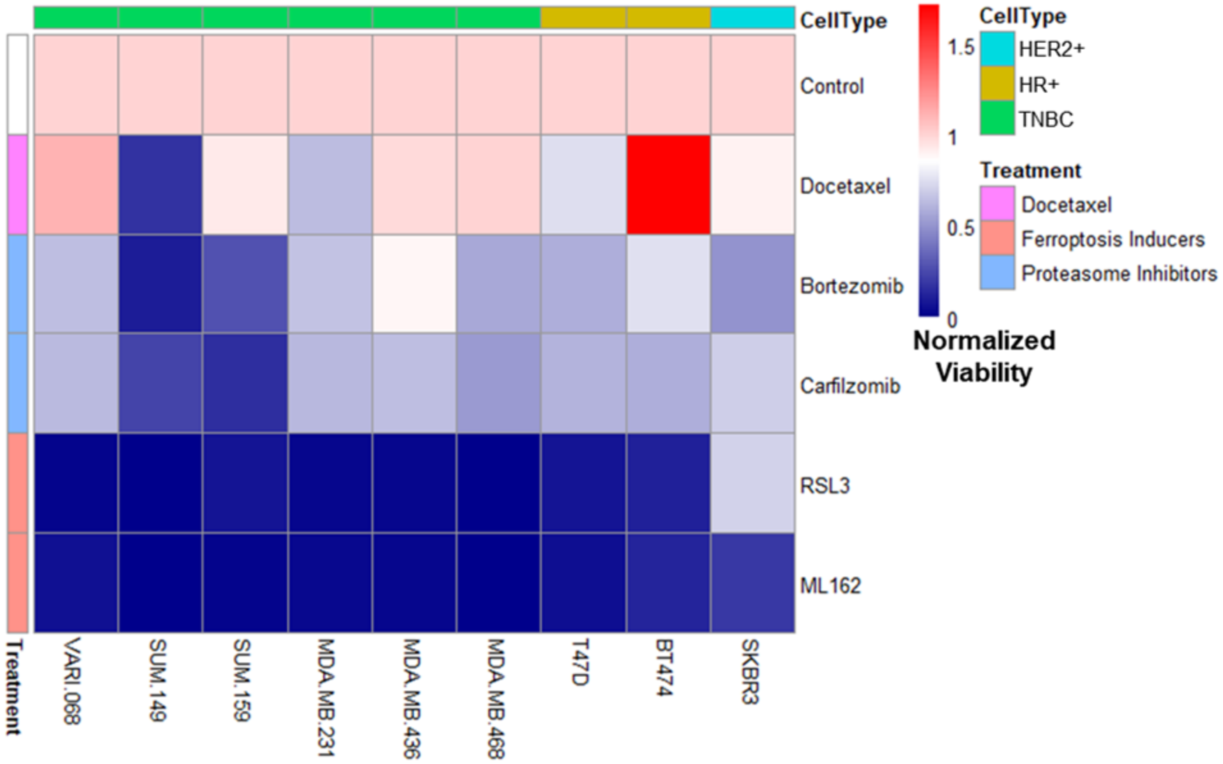

Supplementary Figure 10. Validation of anti-PGCC compounds for non-PGCCs with Paclitaxel treatment. Nine breast cancer cell lines (hormone receptor (HR) positive (T47D and BT474), HER2 positive (SKBR3), and TNBC (Vari068, SUM149, SUM159, MDA-MB-231, MDA-MB-436, and MDA-MB-468) were tested. Color gradient represents the cell viability normalized to the control. Different cell lines treated with Paclitaxel were resistant to Docetaxel yet responded to proteasome inhibitors and ferroptosis inducers. Statistics is provided in Supplementary Table 8.

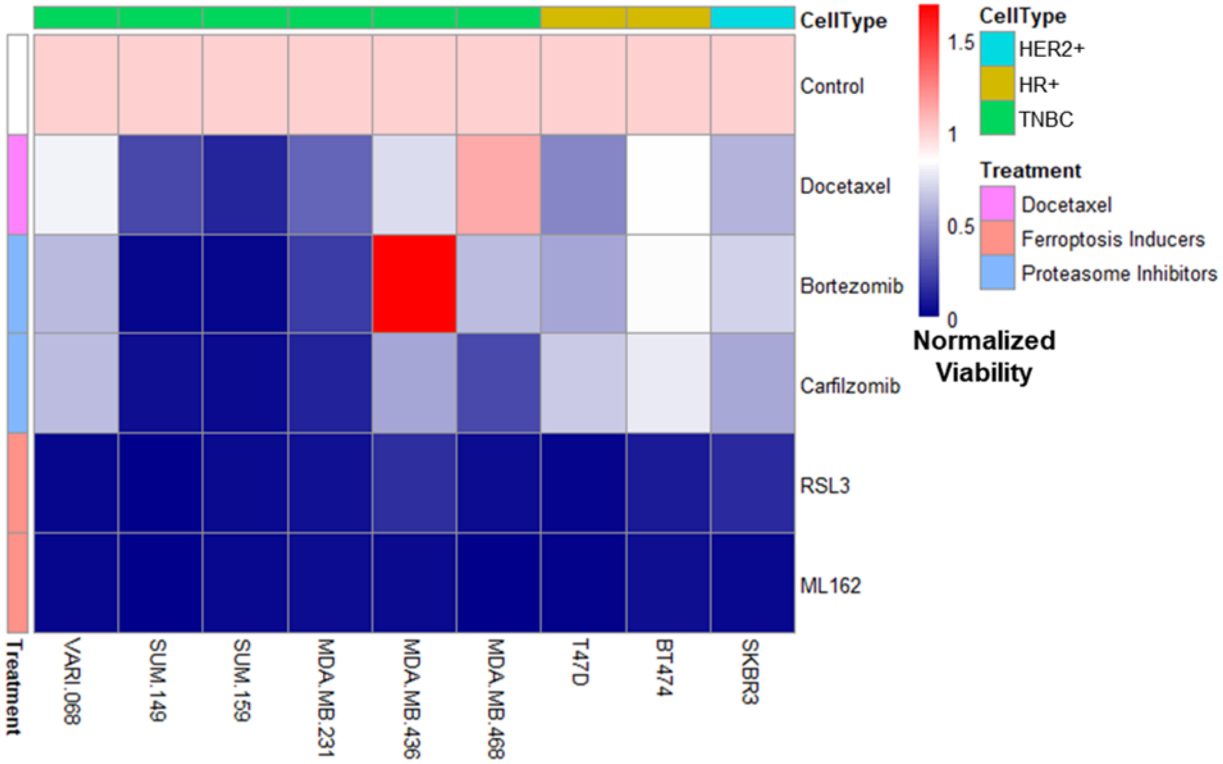

Supplementary Figure 11. Validation of anti-PGCC compounds for non-PGCCs with Alisertib treatment. Nine breast cancer cell lines (hormone receptor (HR) positive (T47D and BT474), HER2 positive (SKBR3), and TNBC (Vari068, SUM149, SUM159, MDA-MB-231, MDA-MB-436, and MDA-MB-468) were tested. Color gradient represents the cell viability normalized to the control. Different cell lines treated with Alisertib were responded to Docetaxel and proteasome inhibitors and ferroptosis inducers. Statistics is provided in Supplementary Table 9.

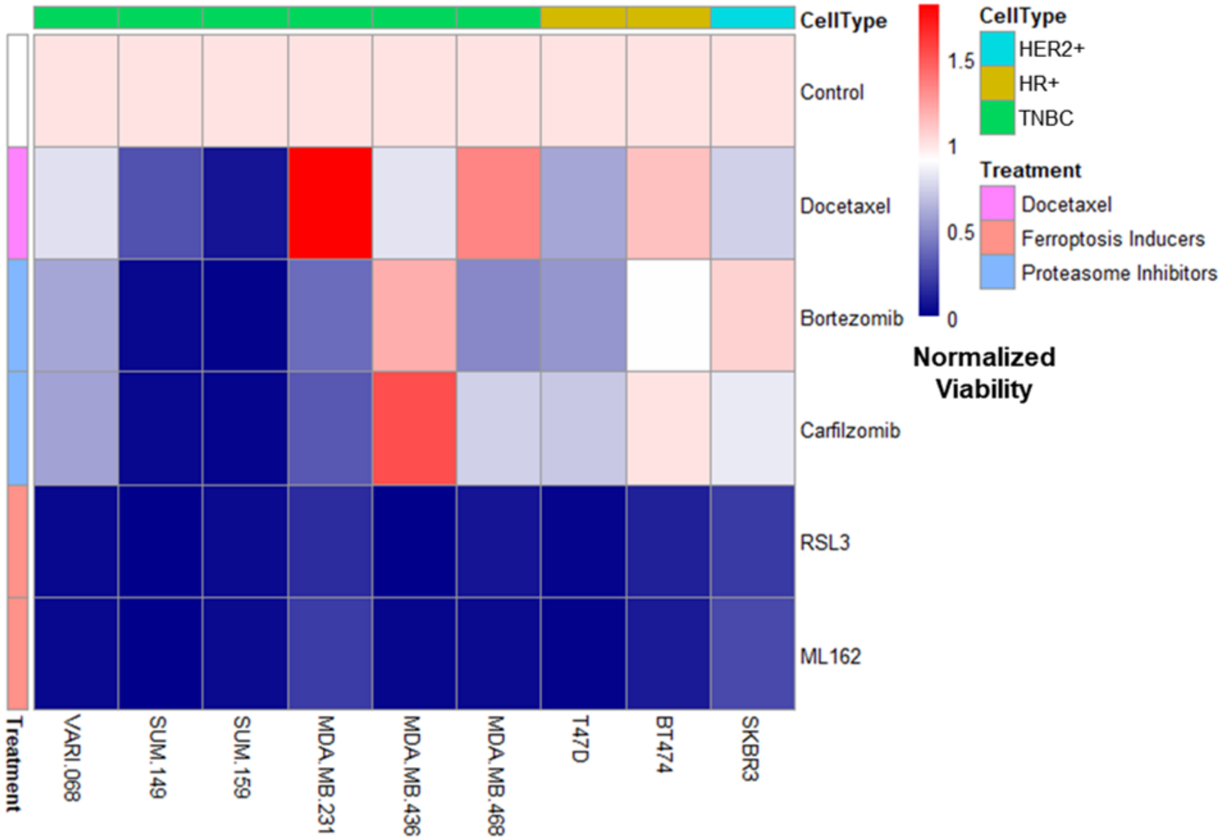

Supplementary Figure 12. Validation of anti-PGCC compounds for non-PGCCs with Tozasertib treatment. Nine breast cancer cell lines (hormone receptor (HR) positive (T47D and BT474), HER2 positive (SKBR3), and TNBC (Vari068, SUM149, SUM159, MDA-MB-231, MDA-MB-436, and MDA-MB-468) were tested. Color gradient represents the cell viability normalized to the control. Different cell lines treated with Tozasertib were responded to Docetaxel and proteasome inhibitors and ferroptosis inducers. Statistics is provided in Supplementary Table 10.

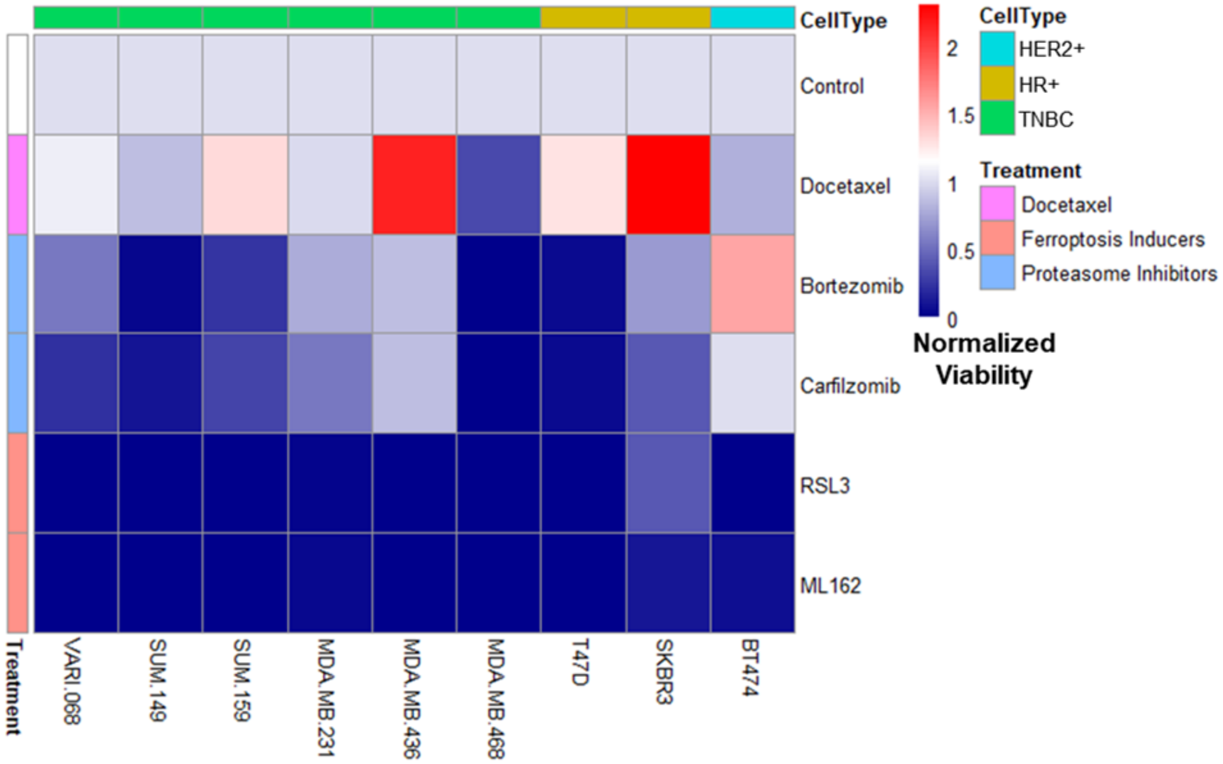

Supplementary Figure 13. Validation of anti-PGCC compounds for PGCCs with Cabazitaxel treatment. Nine breast cancer cell lines (hormone receptor (HR) positive (T47D and BT474), HER2 positive (SKBR3), and TNBC (Vari068, SUM149, SUM159, MDA-MB-231, MDA-MB-436, and MDA-MB-468) were tested. Color gradient represents the cell viability normalized to the control. PGCCs of different cell lines generated by Cabazitaxel were resistant to Docetaxel yet responded to proteasome inhibitors and ferroptosis inducers. Statistics is provided in Supplementary Table 11.

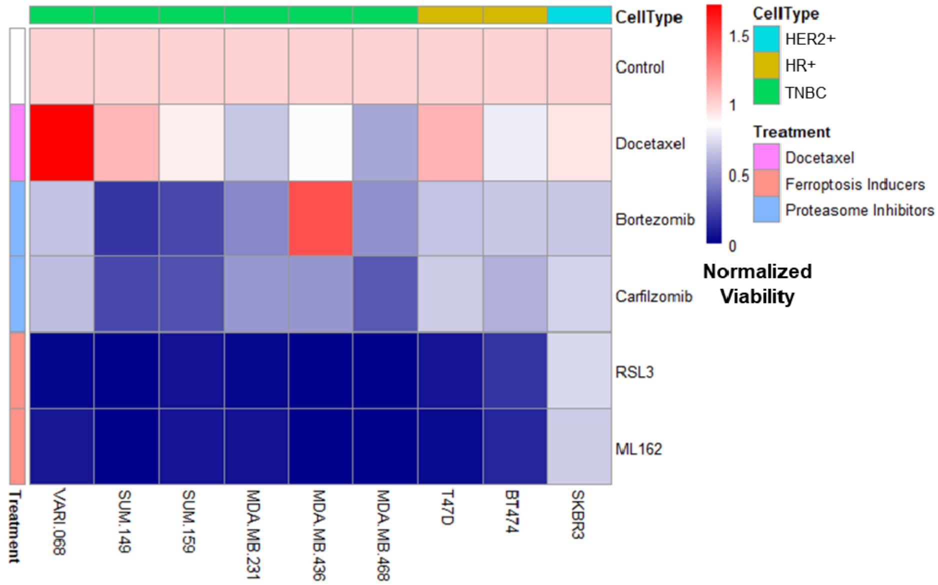

Supplementary Figure 14. Validation of anti-PGCC compounds for non-PGCCs with Cabazitaxel treatment. Nine breast cancer cell lines (hormone receptor (HR) positive (T47D and BT474), HER2 positive (SKBR3), and TNBC (Vari068, SUM149, SUM159, MDA-MB-231, MDA-MB-436, and MDA-MB-468) were tested. Color gradient represents the cell viability normalized to the control. Different cell lines treated with Cabazitaxel were resistant to Docetaxel yet responded to proteasome inhibitors and ferroptosis inducers. Statistics is provided in Supplementary Table 12.

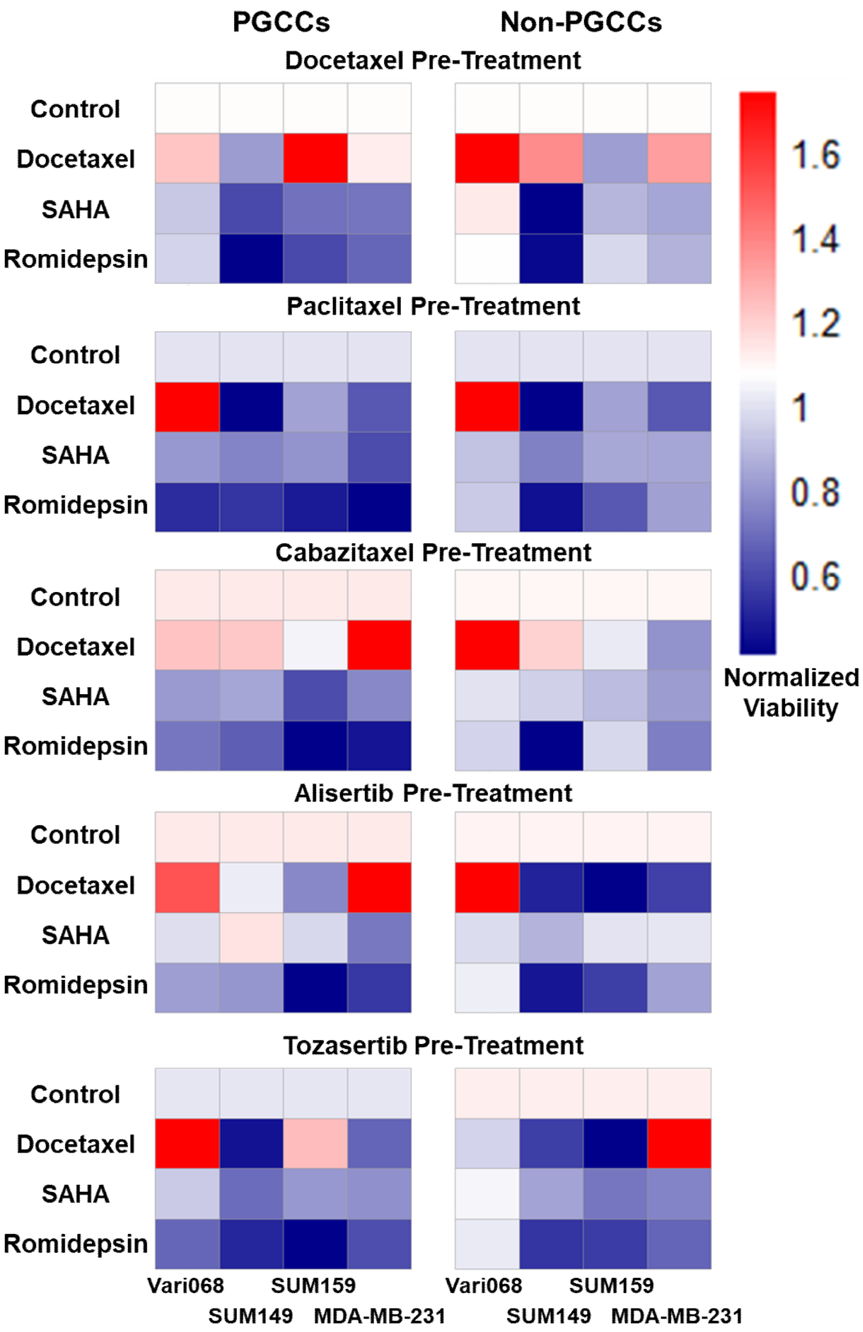

Supplementary Figure 15. Validation of anti-PGCC effects for cell lines with different pre-treatments. Color gradient represents the cell viability normalized to the control. Statistics are provided in Supplementary Table 13-S22.

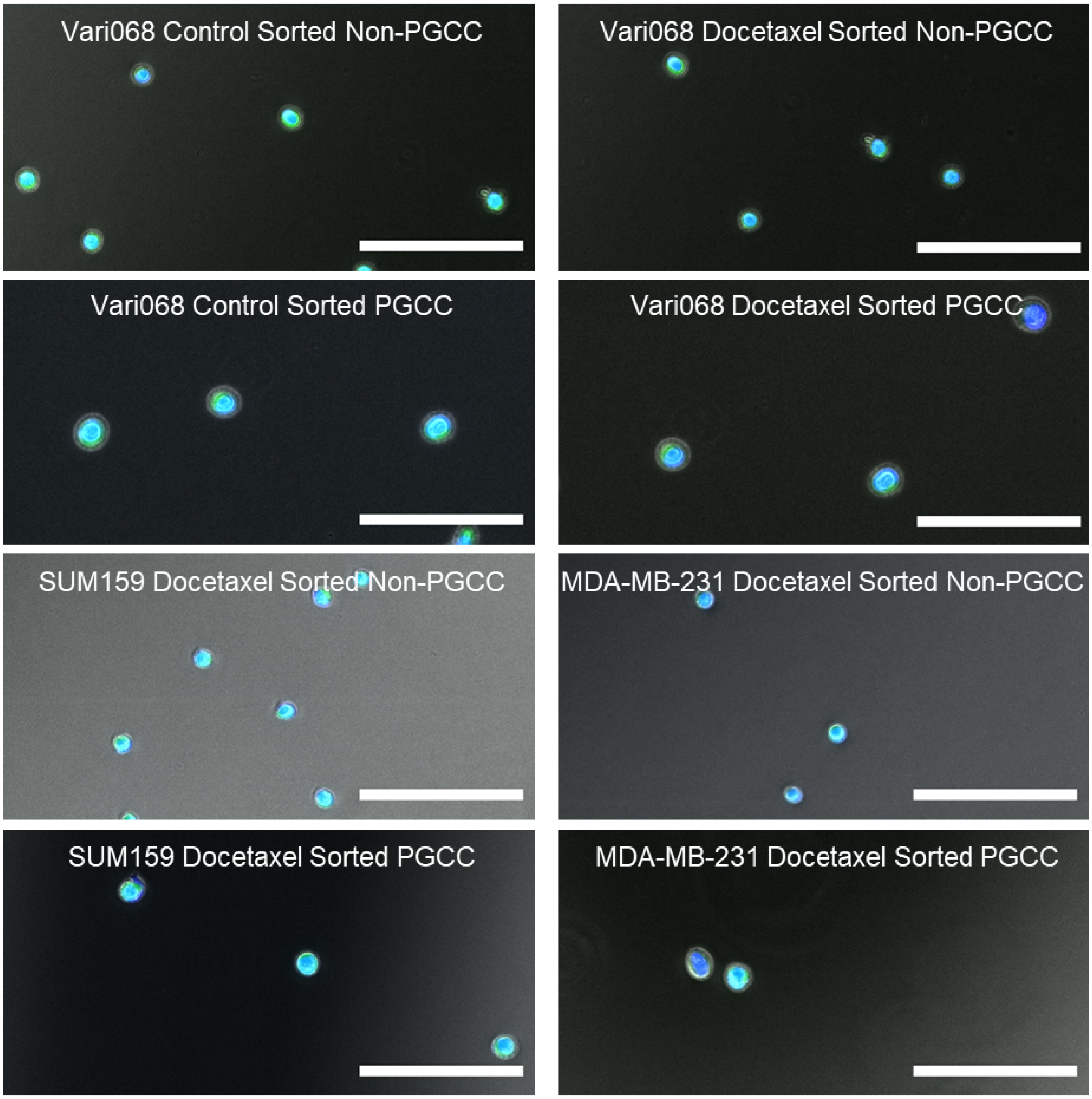

Supplementary Figure 16. Representative images for sorted PGCCs and non-PGCCs. Cells were stained with LIVE (green), Dead (red) and Hoechst (blue) staining. (Scale bar: 100 μm)

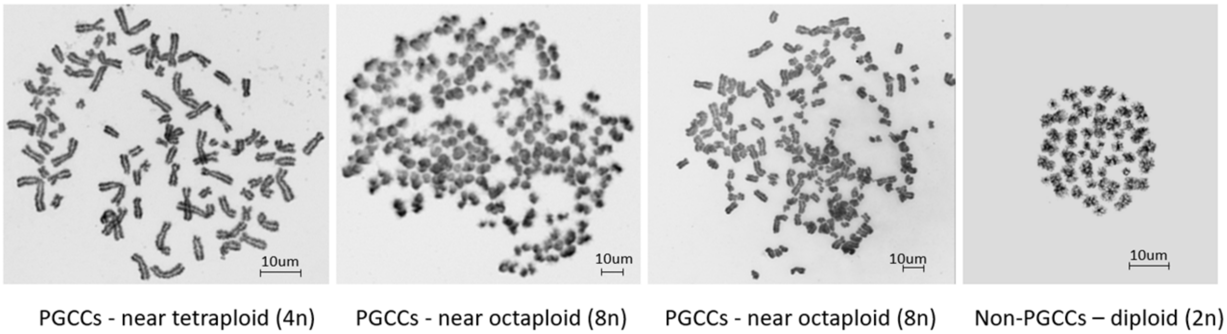

Supplementary Figure 17. karyotyping Docetaxel induced SUM159 PGCCs and non-PGCCs by G-banding metaphase analysis.

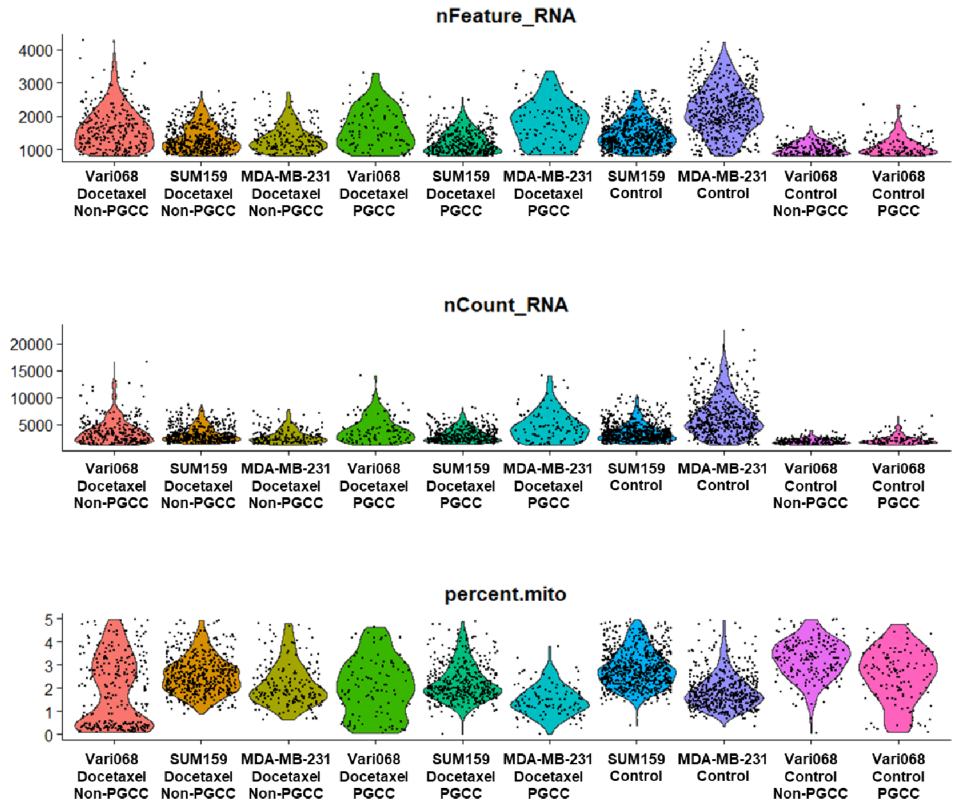

Supplementary Figure 18. The quality control of scRNA-Seq data. The number of genes (nFeature_RNA), the number of transcripts (nCount_RNA), the percentage of mitochondrial genes (percent.mito) of each sample. Each dot represents a cell.

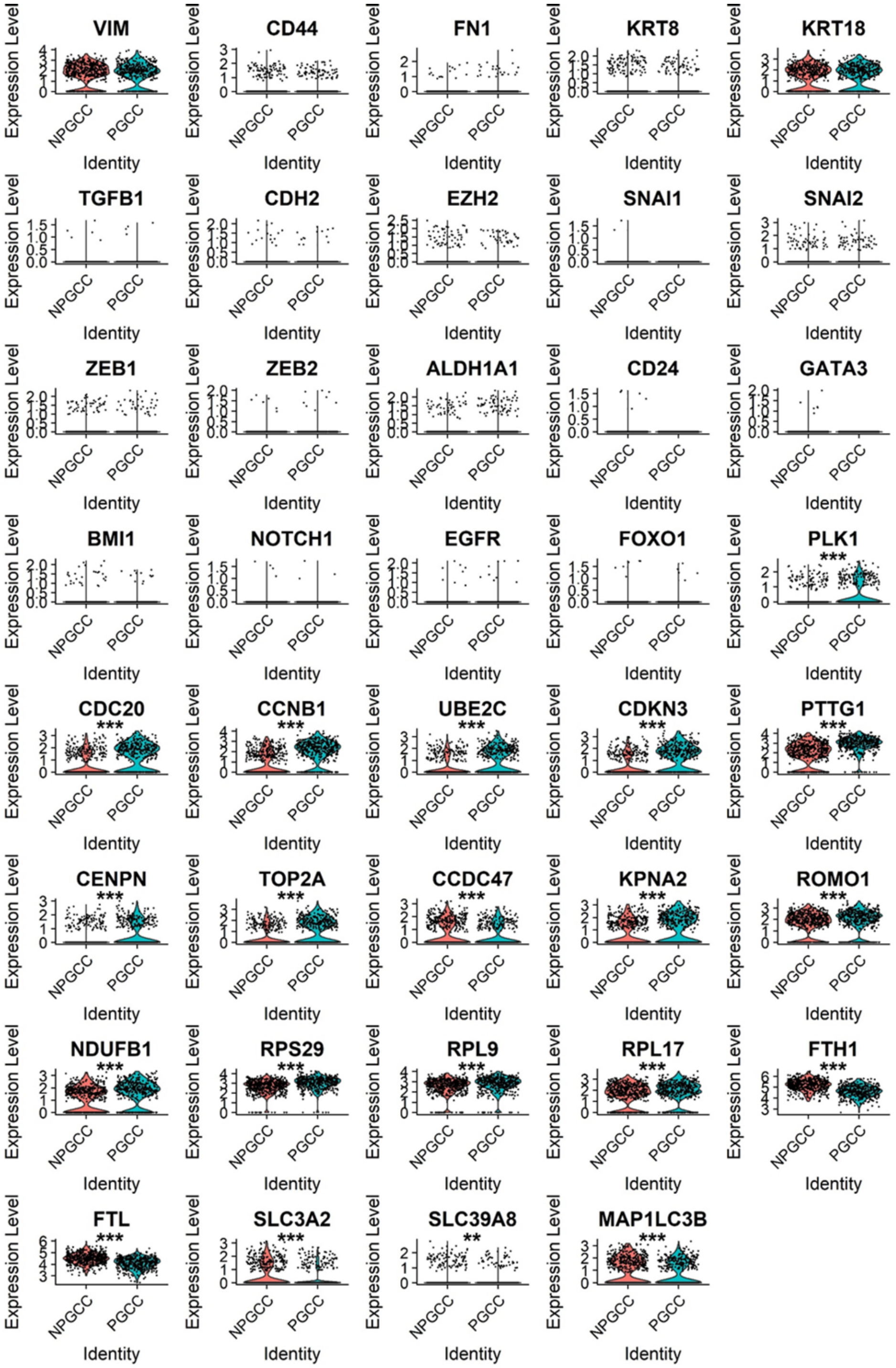

Supplementary Figure 19. Violin plots of Docetaxel treated SUM159 PGCCs and non-PGCCs cells with statistical test. Y-axis represents gene expression with logarithmic scale. Each dot represents one cell. * refers to P < 0.05. ** refers to P < 0.01, and *** refers to P < 0.001.

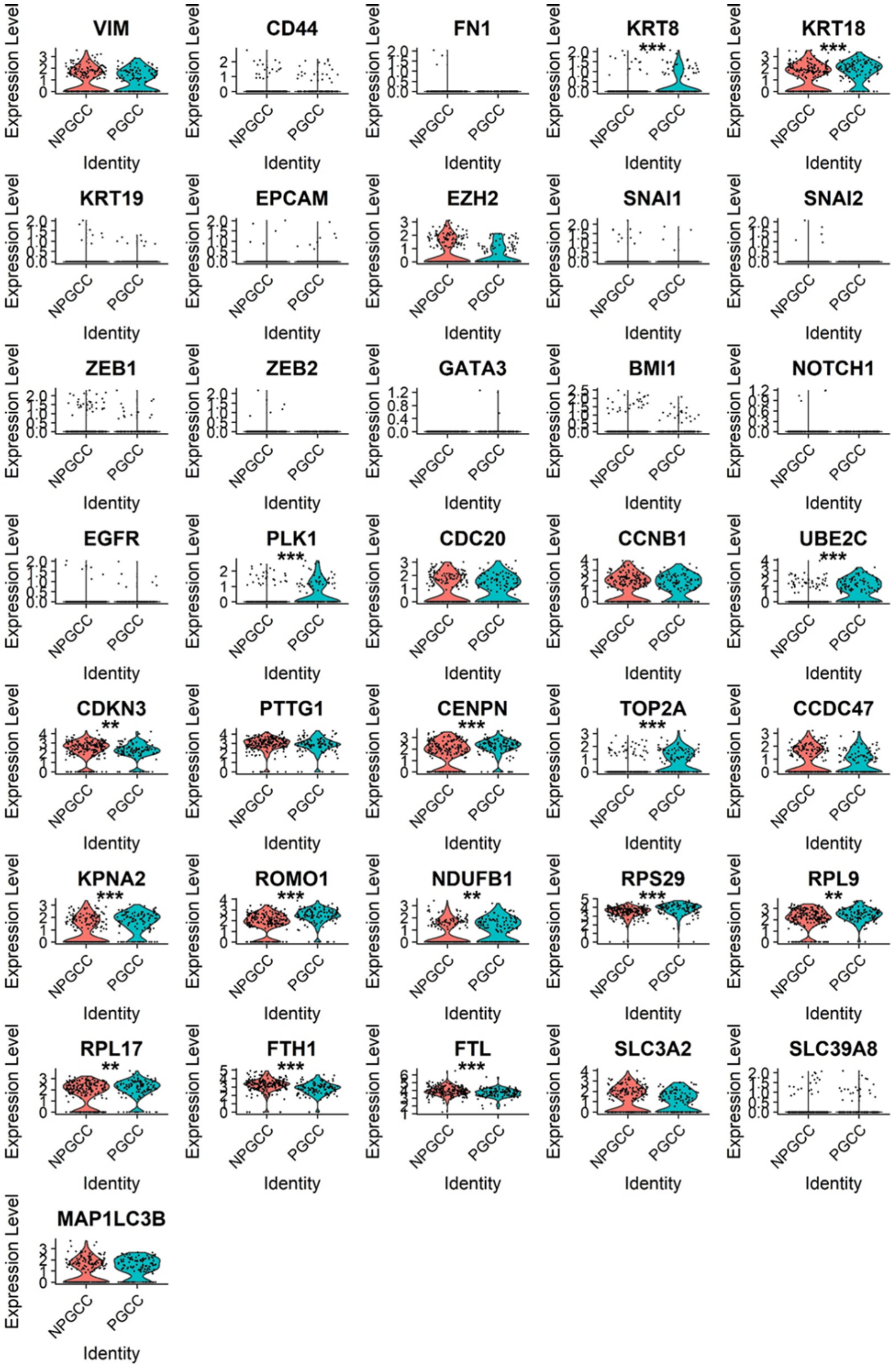

Supplementary Figure 20. Violin plots of Docetaxel treated MDA-MB-231 PGCCs and non-PGCCs cells with statistical test. Y-axis represents gene expression with logarithmic scale. Each dot represents one cell. * refers to P < 0.05. ** refers to P < 0.01, and *** refers to P < 0.001.

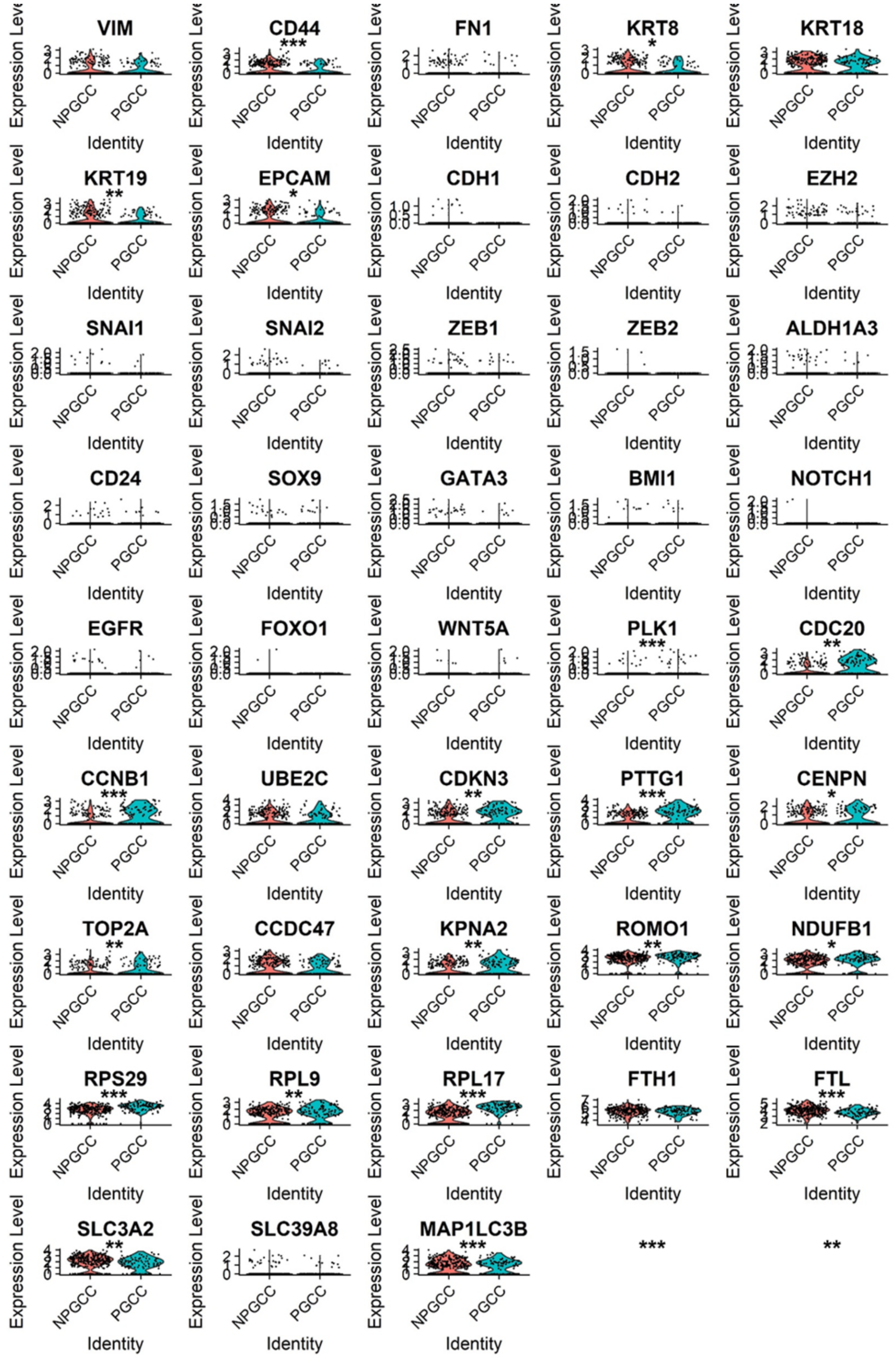

***

**

Supplementary Figure 21. Violin plots of Docetaxel tr[illegible] and non-PGCCs cells with statistical test. Y-axis represents gene expression with logarithmic scale. Each dot represents one cell. * refers to P < 0.05. ** refers to P < 0.01, and *** refers to P < 0.001.

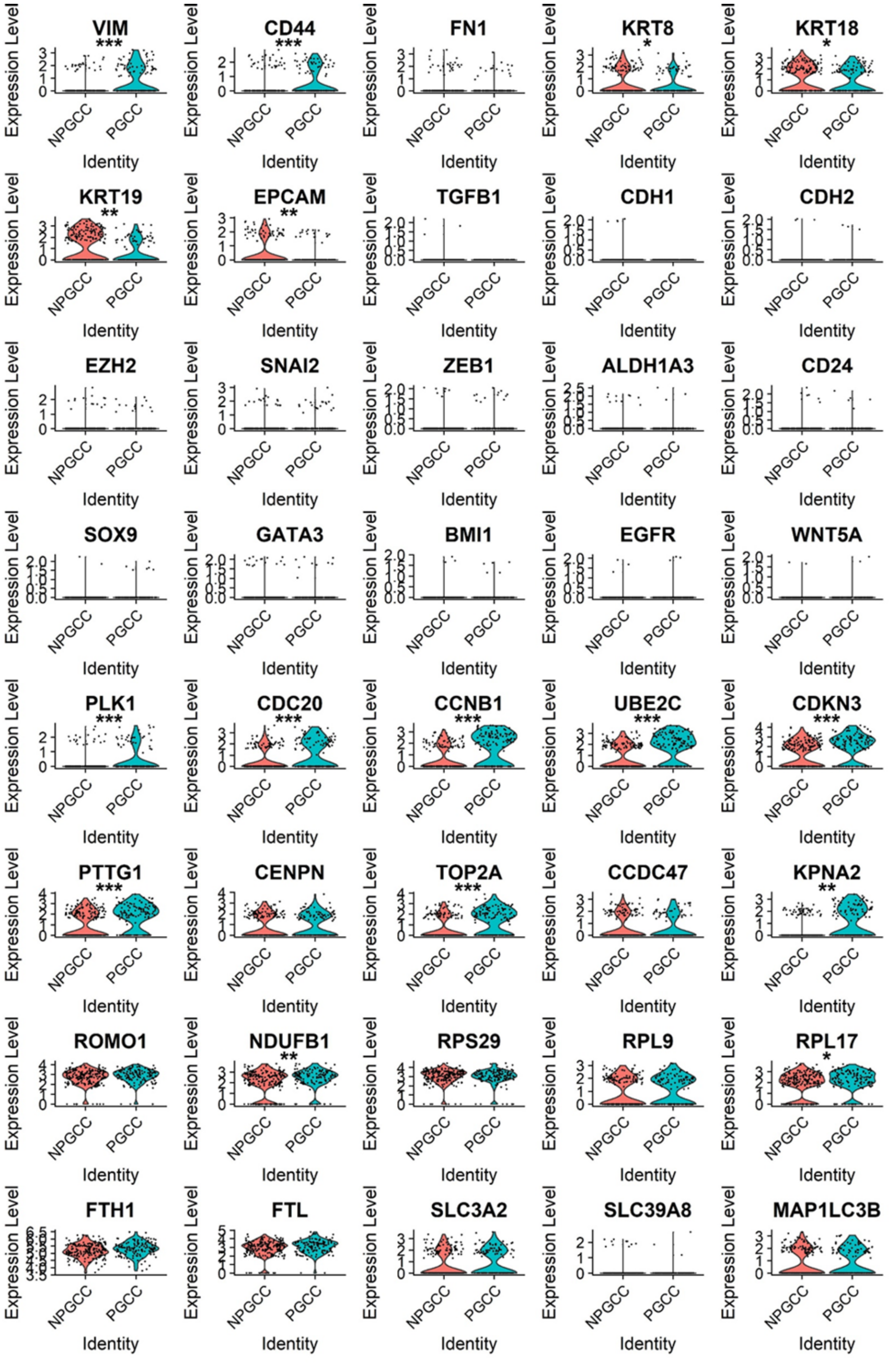

Supplementary Figure 22. Violin plots of untreated Vari068 PGCCs and non-PGCCs cells with statistical test. Y-axis represents gene expression with logarithmic scale. Each dot represents one cell. * refers to P < 0.05. ** refers to P < 0.01, and *** refers to P < 0.001.

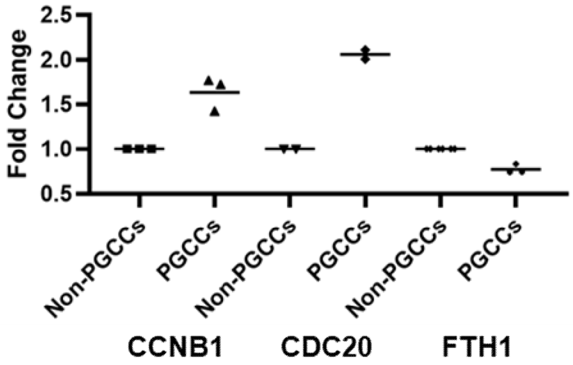

Supplementary Figure 23. Validation of gene expression. Bar graph shows qRT-PCR analysis of CCNB1, CDC20, FTH1 expression in Docetaxel induced SUM159 non-PGCCs and PGCCs. N = 2 for CDC20, and N = 3 for CCNB1 and FTH1.

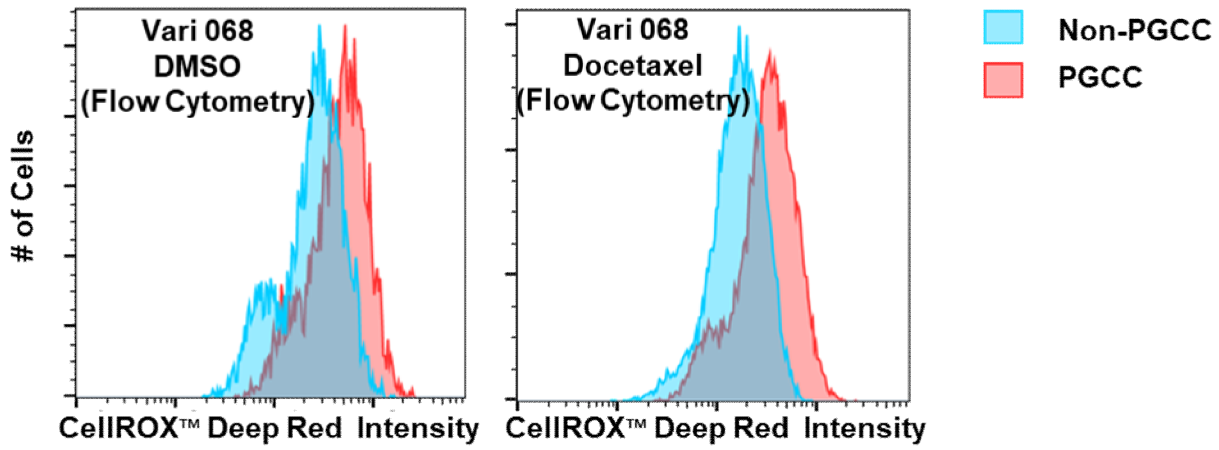

Supplementary Figure 24. ROS level of PGCC and non-PGCC groups. Flow cytometry experiments using Vari068 were performed. PGCCs were found to have significantly higher ROS level with and without the treatment of Docetaxel.

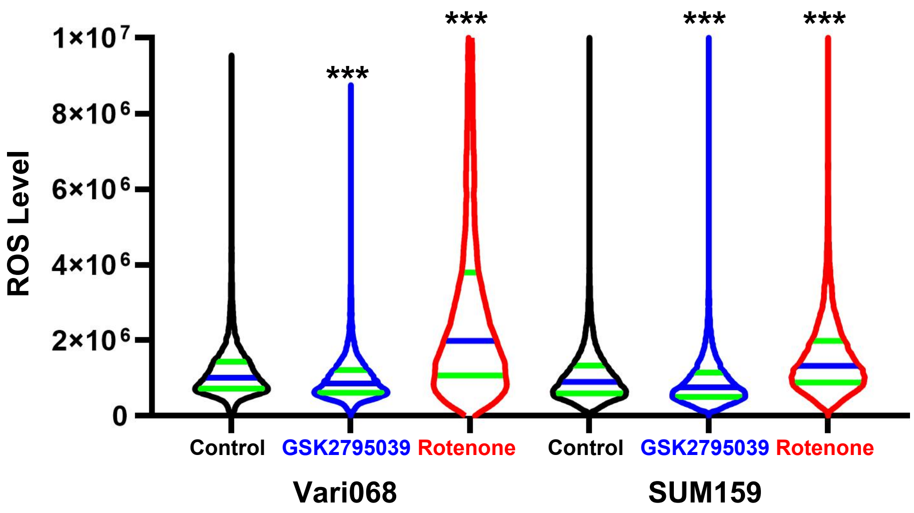

Supplementary Figure 25. ROS level altered with ROS regulators treatment. ROS level of Rotenone groups are higher than DMSO control groups and ROS level of GSK2795039 groups are lower than DMSO control groups both for Vari068 and SUM159. (n = 16,171 cells for Vari068 control, 9,330 cells for Vari068 GSK2795039, 10,119 cells for Vari068 Rotenone and 31,701 cells for SUM159 control, 30,686 cells for SUM159 GSK2795039, 22,699 cells for SUM159 Rotenone) Black curves in violin plot indicate DMSO control, blue curves indicate GSK2795039 treatment, red curves indicate Rotenone treatment. Green lines mean quartiles and blue lines mean median of all cells.

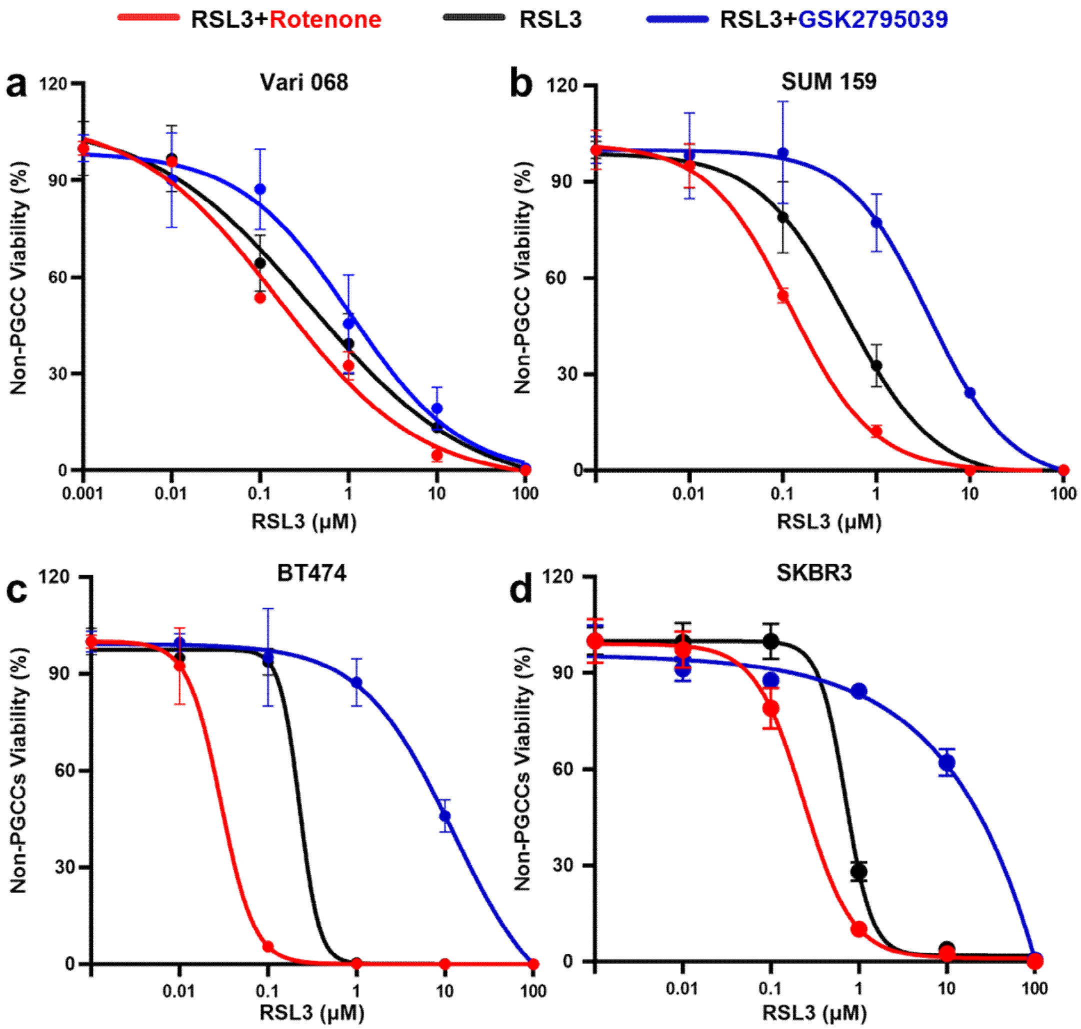

Supplementary Figure 26. Rescue and sensitize non-PGCCs to ferroptosis by altering ROS. 1 μM of Docetaxel was treated 24 h for Vari 068 and SUM 159 cell and 1 μM of Alisertib was treated 24 h for BT474 and SKBR3 cell lines. Rotenone (10 μM for Vari068 and SKBR3, 300 nM for SUM159, 3 μM for BT474) was selected to boost ROS and GSK2795039 (40 μM for Vari068, BT474, and SKBR3, 100 μM for SUM159) to reduce ROS. Compounds efficacy IC50 curves of RSL3 (from 100 μM to 0.001 μM) alone (black), RSL3 with Rotenone (red), and RSL3 with GSK2795039 (blue) were tested for comparison. IC50 values are provided in Supplementary Table 24. Error bars indicate standard error of the mean (SEM). N = 3.

Supplementary Table 1. Molecular information, basic medium and subtype of cell lines in this study.

Cell lines	ER	PR	HER2	Subtype	Base Medium
VARI 068	-	-	-	TNBC	DMEM
SUM 149	-	-	-	TNBC	Ham's F12
SUM 159	-	-	-	TNBC	Ham's F12
MDA MB 231	-	-	-	TNBC	DMEM
MDA MB 436	-	-	-	TNBC	DMEM
MDA MB 468	-	-	-	TNBC	DMEM
T47D	+	+	-	Luminal A	RPMI
BT474	+	+	+	Luminal B	DMEM
SKBR3	-	-	+	HER2	RPMI

Supplementary Table 2 Inhibition statistical results for multi cell lines' PGCCs induced by Docetaxel.

Cell lines	# of PGCCs					
	DMSO	Docetaxel	Bortezomib	Carfilzomib	RSL3	ML162
VARI 068	327	384	80	61	2	1
SUM 149	27	18	3	0	0	0
SUM 159	164	267	24	28	0	0
MDA MB 231	110	116	62	54	3	4
MDA MB 436	6	3	3	3	0	0
MDA MB 468	12	6	6	1	0	0
T47D	21	28	1	6	0	0
BT474	12	26	3	4	0	2
SKBR3	47	17	30	32	1	2
Mean	80.67	96.11	23.56	21	0.6667	1
95% CI		-72.41, 41.52	0.1482, 114.1	2.704, 116.6	23.04, 137.0	22.70, 136.6
P Value		0.5868	<0.05	<0.05	<0.05	<0.05

Supplementary Table 3 Inhibition statistical results for multi cell lines' PGCCs induced by Paclitaxel.

	# of PGCCs					
Cell lines	DMSO	Docetaxel	Bortezomib	Carfilzomib	RSL3	ML162
VARI 068	349	396	45	38	2	0
SUM 149	42	17	3	1	0	0
SUM 159	117	97	5	4	0	0
MDA MB 231	63	40	20	31	1	3
MDA MB 436	19	12	7	5	0	0
MDA MB 468	11	5	1	1	0	0
T47D	12	16	0	3	0	0
BT474	18	13	4	4	1	4
SKBR3	29	14	55	32	1	0
Mean	73.33	67.78	15.56	13.22	0.5556	0.7778
95% CI		-50.78, 61.89	1.446, 114.1	3.779, 116.4	16.45, 129.1	16.22, 128.9
P Value		0.843	<0.05	<0.05	<0.05	<0.05

Supplementary Table 4 Inhibition statistical results for multi cell lines' PGCCs induced by Alisertib.

Cell lines	# of PGCCs					
	DMSO	Docetaxel	Bortezomib	Carfilzomib	RSL3	ML162
VARI 068	357	513	307	165	3	1
SUM 149	530	467	13	23	0	0
SUM 159	576	339	67	93	20	9
MDA MB 231	694	686	164	119	85	14
MDA MB 436	13	19	11	27	1	1
MDA MB 468	19	13	2	9	0	0
T47D	59	197	10	10	0	0
BT474	140	73	18	97	2	0
SKBR3	174	108	185	108	0	0
Mean	284.7	268.3	86.33	72.33	12.33	2.778
95% CI		-105.3, 137.9	76.73, 319.9	90.73, 333.9	150.7, 393.9	160.3, 403.5
P Value		0.7874	<0.05	<0.05	<0.05	<0.05

Supplementary Table 5 Inhibition statistical results for multi cell lines' PGCCs induced by Tozasertib.

Cell lines	# of PGCCs					
	DMSO	Docetaxel	Bortezomib	Carfilzomib	RSL3	ML162
VARI 068	619	636	325	193	7	1
SUM 149	423	486	27	21	2	0
SUM 159	529	215	46	78	116	6
MDA MB 231	681	672	107	80	156	35
MDA MB 436	19	11	11	8	1	0
MDA MB 468	16	9	6	2	0	0
T47D	64	78	6	2	0	0
BT474	238	155	17	45	10	3
SKBR3	224	121	150	96	0	5
Mean	312.6	264.8	77.22	58.33	32.44	5.556
95% CI		-72.84, 168.4	114.7, 355.9	133.6, 374.8	159.5, 400.7	186.4, 427.6
P Value		0.4281	<0.05	<0.05	<0.05	<0.05

Supplementary Table 6 Inhibition statistical results for multi cell lines' non-PGCCs with Docetaxel treatment.

Cell lines	# of Non-PGCCs					
	DMSO	Docetaxel	Bortezomib	Carfilzomib	RSL3	ML162
VARI 068	579	637	233	184	16	18
SUM 149	14	19	3	3	0	0
SUM 159	115	79	65	74	7	10
MDA MB 231	124	161	75	59	8	14
MDA MB 436	31	30	24	20	3	1
MDA MB 468	31	13	7	6	0	0
T47D	571	460	278	224	27	34
BT474	178	123	131	130	43	31
SKBR3	171	216	128	139	153	128
Mean	201.6	193.1	104.9	93.22	28.56	26.22
95% CI		-86.6, 103.5	1.624, 191.7	13.29, 203.4	77.96, 268	80.29, 270.4
P Value		0.8584	0.0464	0.0265	0.0007	0.0006

Supplementary Table 7 Inhibition statistical results for multi cell lines' non-PGCCs with Paclitaxel treatment.

Cell lines	# of Non-PGCCs					
	DMSO	Docetaxel	Bortezomib	Carfilzomib	RSL3	ML162
VARI 068	540	598	345	339	8	30
SUM 149	101	17	10	23	0	0
SUM 159	100	93	28	16	7	2
MDA MB 231	185	117	121	115	4	5
MDA MB 436	44	43	39	28	1	1
MDA MB 468	23	23	13	12	0	0
T47D	601	449	353	364	43	29
BT474	221	246	167	129	25	28
SKBR3	259	233	129	180	182	50
Mean	230.4	202.1	133.9	134	30	16.11
95% CI		-59.7, 116.4	8.521, 184.6	8.41, 184.5	112.4, 288.5	126.3, 302.4
P Value		0.5191	0.0324	0.0326	<0.0001	<0.0001

Supplementary Table 8 Inhibition statistical results for multi cell lines' non-PGCCs with Alisertib treatment.

Cell lines	# of Non-PGCCs					
	DMSO	Docetaxel	Bortezomib	Carfilzomib	RSL3	ML162
VARI 068	822	658	508	511	19	18
SUM 149	1210	289	30	58	0	1
SUM 159	1461	180	31	55	53	48
MDA MB 231	1201	402	238	136	69	54
MDA MB 436	51	37	86	28	8	2
MDA MB 468	24	27	15	6	1	0
T47D	1223	541	673	814	21	9
BT474	1029	873	858	797	91	49
SKBR3	923	550	643	512	128	30
Mean	882.7	395.2	342.4	324.1	43.33	23.44
95% CI		250.2, 724.7	303, 777.5	321.3, 795.8	602.1, 1077	622, 1096
P Value		0.0002	<0.0001	<0.0001	<0.0001	<0.0001

Supplementary Table 9 Inhibition statistical results for multi cell lines' non-PGCCs with Tozasertib treatment.

Cell lines	# of Non-PGCCs					
	DMSO	Docetaxel	Bortezomib	Carfilzomib	RSL3	ML162
VARI 068	769	612	455	445	27	24
SUM 149	1062	309	36	36	1	1
SUM 159	1794	136	23	29	68	71
MDA MB 231	271	491	104	86	44	58
MDA MB 436	47	38	56	72	0	1
MDA MB 468	27	36	13	20	2	1
T47D	1023	603	544	728	19	10
BT474	685	772	623	686	78	67
SKBR3	680	504	723	565	140	179
Mean	706.4	389	286.3	296.3	42.11	45.78
95% CI		60.72, 574.2	163.4, 676.8	153.4, 666.8	407.6, 921.1	403.9, 917.4
P Value		0.0167	0.002	0.0025	<0.0001	<0.0001

Supplementary Table 10 Inhibition statistical results for multi cell lines' PGCCs induced by Cabazitaxel.

Cell lines	# of PGCCs					
	DMSO	Docetaxel	Bortezomib	Carfilzomib	RSL3	ML162
VARI 068	330	354	179	72	1	1
SUM 149	144	124	5	14	0	0
SUM 159	115	151	27	36	0	0
MDA MB 231	75	74	58	41	2	3
MDA MB 436	7	15	6	6	0	0
MDA MB 468	6	2	0	0	0	0
T47D	22	28	1	1	0	0
BT474	10	23	7	4	4	1
SKBR3	224	121	150	96	0	5
Mean	88.63	96.38	22.88	21.75	0.875	0.625
95% CI		-63.19, 47.69	10.31, 121.2	11.44, 122.3	32.31, 143.2	32.56, 143.4
P Value		0.7782	<0.05	<0.05	<0.05	<0.05

Supplementary Table 11 Inhibition statistical results for multi cell lines' non-PGCCs with Cabazitaxel treatment.

Cell lines	# of Non-PGCCs					
	DMSO	Docetaxel	Bortezomib	Carfilzomib	RSL3	ML162
VARI 068	576	637	373	365	15	48
SUM 149	111	122	20	28	0	0
SUM 159	112	102	28	31	7	8
MDA MB 231	215	142	98	110	6	14
MDA MB 436	33	39	23	22	2	0
MDA MB 468	27	15	13	8	0	0
T47D	579	639	381	396	40	21
BT474	270	213	180	160	48	35
SKBR3	244	229	163	171	177	166
Mean	240.8	237.6	142.1	143.4	32.78	32.44
95% CI		-92.45, 98.9	2.991, 194.3	1.657, 193	112.3, 303.7	112.7, 304
P Value		0.9461	0.0436	0.0463	<0.0001	<0.0001

Supplementary Table 12 Inhibition statistical results for multi cell lines' PGCCs with Cabazitaxel treatment.

Treatment	# of PGCCs				Mean	95% CI	P Value
	VARI 068	SUM 149	SUM 159	MDA MB 231			
DMSO	210	33	93	173	127.3		
Docetaxel	246	22	170	182	155.3	-68.11, 12.09	0.1485
Vorinostat (SAHA)	170	13	49	93	81.25	5.902, 86.1	0.029
Romidepsin	178	5	37	85	76.25	10.9, 91.1	0.0183

Supplementary Table 13 Inhibition statistical results for multi cell lines' non-PGCCs with Cabazitaxel treatment.

Treatment	# of Non-PGCCs				Mean	95% CI	P Value
	VARI 068	SUM 149	SUM 159	MDA MB 231			
DMSO	503	77	105	368	263.3		
Docetaxel	905	104	72	477	389.5	-289.3, 36.79	0.1137
Vorinostat (SAHA)	432	14	80	260	196.5	-96.29,229.8	0.3785
Romidepsin	447	16	91	276	207.5	-107.3,218.8	0.459

Supplementary Table 14 Inhibition statistical results for multi cell lines' PGCCs with Cabazitaxel treatment.

Treatment	# of PGCCs				Mean	95% CI	P Value
	VARI 068	SUM 149	SUM 159	MDA MB 231			
DMSO	246	34	96	186	140.5		
Docetaxel	292	20	63	129	125.9	-35.72, 64.84	0.5288
Vorinostat (SAHA)	156	11	41	91	74.75	15.47, 116	0.016
Romidepsin	141	8	38	89	69	21.22, 121.8	0.0105

Supplementary Table 15 Inhibition statistical results for multi cell lines' non-PGCCs with Cabazitaxel treatment.

Treatment	# of Non-PGCCs				Mean	95% CI	P Value
	VARI 068	SUM 149	SUM 159	MDA MB 231			
DMSO	428	51	162	225	216.5		
Docetaxel	510	21	134	135	200	-50.98, 83.88	0.5944
Vorinostat (SAHA)	328	27	97	135	146.8	2.32, 137.2	0.044
Romidepsin	342	23	103	125	148.3	0.8202, 135.7	0.0478

Supplementary Table 16 Inhibition statistical results for multi cell lines' PGCCs with Cabazitaxel treatment.

Treatment	# of PGCCs				Mean	95% CI	P Value
	VARI 068	SUM 149	SUM 159	MDA MB 231			
DMSO	251	49	161	95	139		
Docetaxel	278	54	147	158	159	-63.18, 23.28	0.3237
Vorinostat (SAHA)	165	34	71	58	82	13.77, 100.2	0.0154
Romidepsin	140	24	36	27	56.75	39.02, 125.5	0.002

Supplementary Table 17 Inhibition statistical results for multi cell lines' non-PGCCs with Cabazitaxel treatment.

Treatment	# of Non-PGCCs				Mean	95% CI	P Value
	VARI 068	SUM 149	SUM 159	MDA MB 231			
DMSO	487	134	86	204	227.8		
Docetaxel	530	147	78	135	222.6	-40, 50.34	0.8017
Vorinostat (SAHA)	433	82	51	141	176.8	5.831, 96.17	0.031
Romidepsin	414	32	62	123	157.8	24.83, 115.2	0.0067

Supplementary Table 18 Inhibition statistical results for multi cell lines' PGCCs with Cabazitaxel treatment.

Treatment	# of PGCCs				Mean	95% CI	P Value
	VARI 068	SUM 149	SUM 159	MDA MB 231			
DMSO	332	114	226	476	287		
Docetaxel	247	91	112	597	261.8	-127.5, 178	0.7171
Vorinostat (SAHA)	208	81	64	182	133.8	0.4952, 306	0.0494
Romidepsin	231	72	43	179	131.3	2.995, 308.5	0.0465

Supplementary Table 19 Inhibition statistical results for multi cell lines' non-PGCCs with Cabazitaxel treatment.

Treatment	# of Non-PGCCs				Mean	95% CI	P Value
	VARI 068	SUM 149	SUM 159	MDA MB 231			
DMSO	594	318	151	629	423		
Docetaxel	669	76	79	211	258.6	11.72, 317	0.0376
Vorinostat (SAHA)	502	67	32	452	263.3	7.094, 312.4	0.0421
Romidepsin	514	73	46	415	262	8.344, 313.7	0.0408

Supplementary Table 20 Inhibition statistical results for multi cell lines' PGCCs with Cabazitaxel treatment.

Treatment	# of PGCCs				Mean	95% CI	P Value
	VARI 068	SUM 149	SUM 159	MDA MB 231			
DMSO	412	181	161	358	278		
Docetaxel	537	73	203	227	260.1	-83, 118.8	0.6975
Vorinostat (SAHA)	280	79	76	212	161.8	15.34, 217.2	0.0285
Romidepsin	266	84	57	207	153.5	23.59, 225.4	0.021

Supplementary Table 21 Inhibition statistical results for multi cell lines' non-PGCCs with Cabazitaxel treatment.

Treatment	# of Non-PGCCs				Mean	95% CI	P Value
	VARI 068	SUM 149	SUM 159	MDA MB 231			
DMSO	635	281	176	480	393		
Docetaxel	451	68	53	502	268.5	5.688, 243.3	0.0419
Vorinostat (SAHA)	512	78	84	254	232	42.19, 279.8	0.0135
Romidepsin	498	72	50	202	205.5	68.69, 306.3	0.006

Supplementary Table 22. Top-ranked altered pathways (KEGG 2021 Human pathway database) of PGCCs versus non-PGCCs using breast cancer cell lines (SUM159 and MDA-MB-231), patient-derived cells (Vari068), and the overlapping markers between three cell lines.

**Overlapping Markers of Three Cell Lines**	**P-value**	**SUM159**	**P-value**
**Ribosome**	1.91E-05	**Oxidative phosphorylation**	2.79E-09
Coronavirus disease	1.61E-04	Parkinson disease	3.06E-09
**Cell cycle**	9.41E-04	**Cell cycle**	7.13E-09
**Oxidative phosphorylation**	1.22E-03	**Ribosome**	2.25E-07
DNA replication	7.05E-03	Prion disease	1.40E-06
**Ferroptosis**	9.07E-03	p53 signaling pathway	2.80E-06
Parkinson disease	1.13E-02	Amyotrophic lateral sclerosis	2.93E-06
Nucleotide excision repair	1.18E-02	Oocyte meiosis	3.25E-06
Prion disease	1.54E-02	RNA transport	9.29E-06
Spliceosome	1.57E-02	Spliceosome	1.70E-05
Mineral absorption	1.88E-02	Huntington disease	2.71E-05
Protein processing in endoplasmic reticulum	2.21E-02	Alzheimer disease	3.76E-05
Huntington disease	2.24E-02	Coronavirus disease	1.20E-04
p53 signaling pathway	2.71E-02	Thermogenesis	1.20E-04
Riboflavin metabolism	2.77E-02	**Ferroptosis**	1.97E-04
**MDA-MB-231**	**P-value**	**Vari068**	**P-value**
**Ribosome**	3.03E-15	**Ribosome**	1.94E-14
Spliceosome	1.05E-14	Amyotrophic lateral sclerosis	1.52E-12

Parkinson disease	2.83E-12	Parkinson disease	3.68E-12
Amyotrophic lateral sclerosis	3.33E-12	Protein processing in endoplasmic reticulum	7.18E-12
Huntington disease	1.08E-11	Spliceosome	2.13E-09
**Cell cycle**	3.36E-11	Pathways of neurodegeneration	1.27E-08
Protein processing in endoplasmic reticulum	3.70E-10	**Oxidative phosphorylation**	7.89E-08
**Oxidative phosphorylation**	1.09E-09	Huntington disease	1.55E-07
RNA transport	4.57E-09	**Cell cycle**	2.26E-07
Prion disease	2.38E-08	Coronavirus disease	2.97E-07
Ubiquitin mediated proteolysis	2.45E-06	Alzheimer disease	3.14E-07
Alzheimer disease	6.41E-06	Prion disease	5.47E-07
Diabetic cardiomyopathy	9.95E-06	Thermogenesis	5.47E-06
Thermogenesis	1.17E-05	RNA degradation	4.45E-05
Pathways of neurodegeneration	1.77E-05	p53 signaling pathway	5.85E-05

Supplementary Table 23. Top-ranked altered pathways (KEGG 2021 Human pathway database) of untreated PGCCs versus non-PGCCs of Vari068 breast cancer cells.

**Vari068**	**P-value**
**Cell cycle**	1.99E-17
**Ribosome**	4.29E-13
Parkinson disease	1.10E-10
Amyotrophic lateral sclerosis	7.91E-10
Huntington disease	1.98E-09
Alzheimer disease	3.68E-09
Prion disease	6.21E-09
Coronavirus disease	2.58E-08
p53 signaling pathway	3.59E-08
Pathways of neurodegeneration	5.42E-08
DNA replication	1.90E-07
Spliceosome	4.67E-06
Oocyte meiosis	1.62E-05
Pyruvate metabolism	2.80E-05
Cellular senescence	2.88E-05

Supplementary Table 24. Top-ranked altered pathways (NCI-Nature pathway database) of PGCCs versus non-PGCCs using breast cancer cell lines (SUM159 and MDA-MB-231), patient-derived cells (Vari068), and the overlapping markers between three cell lines.

**Overlapping Markers of Three Cell Lines**	**P-value**	**SUM159**	**P-value**
p73 transcription factor network	1.61E-04	**PLK1 signaling events**	3.96E-07
Validated transcriptional targets of TAp63 isoforms	8.49E-04	**Aurora B signaling**	1.21E-06
E2F transcription factor network	2.06E-03	**FOXM1 transcription factor network**	1.48E-06
Signaling events mediated by PRL	2.91E-03	p73 transcription factor network	5.17E-06
**Signaling events mediated by HDAC Class III**	3.72E-03	**Aurora A signaling**	3.85E-05
**FOXM1 transcription factor network**	8.64E-03	Validated targets of C-MYC transcriptional activation	2.47E-04
Signaling events mediated by TCPTP	9.50E-03	Validated transcriptional targets of TAp63 isoforms	8.15E-04
Validated transcriptional targets of deltaNp63 isoforms	1.09E-02	E2F transcription factor network	3.98E-03
Calcineurin-regulated NFAT-dependent transcription in lymphocytes	1.13E-02	ATR signaling pathway	8.76E-03
**PLK1 signaling events**	1.13E-02	Validated targets of C-MYC transcriptional repression	1.02E-02
Validated targets of C-MYC	2.06E-02	Signaling events mediated by PRL	1.18E-02
Regulation of cytoplasmic and nuclear SMAD2/3 signaling	5.79E-02	Fanconi anemia pathway	1.67E-02
Direct p53 effectors	8.22E-02	FoxO family signaling	1.79E-02
IGF1 pathway	9.36E-02	Direct p53 effectors	2.46E-02
**Aurora A signaling**	1.03E-01	p53 pathway	3.14E-02
**MDA-MB-231**	**P-value**	**Vari068**	**P-value**
**PLK1 signaling events**	1.52E-05	**PLK1 signaling events**	9.78E-09

E2F transcription factor network	1.68E-04	Lissencephaly gene (LIS1) in neuronal migration and development	8.44E-04
**Aurora B signaling**	3.52E-04	Integrin-linked kinase signaling	1.25E-03
**FOXM1 transcription factor network**	4.39E-04	p73 transcription factor network	1.40E-03
Signaling events mediated by PRL	9.01E-04	**Aurora B signaling**	1.64E-03
**Aurora A signaling**	1.30E-03	**FOXM1 transcription factor network**	1.98E-03
Validated targets of C-MYC transcriptional activation	1.49E-03	**Signaling events mediated by HDAC Class II**	2.58E-03
Validated targets of C-MYC transcriptional repression	1.68E-03	Validated targets of C-MYC transcriptional activation	4.50E-03
**Signaling events mediated by HDAC Class II**	2.47E-03	Sumoylation by RanBP2 regulates transcriptional repression	6.14E-03
AP-1 transcription factor network	3.72E-03	Validated nuclear estrogen receptor alpha network	6.18E-03
BARD1 signaling events	3.91E-03	ErbB1 downstream signaling	2.03E-02
Integrin-linked kinase signaling	4.41E-03	Direct p53 effectors	2.05E-02
Direct p53 effectors	9.40E-03	Signaling events mediated by HDAC Class I	2.07E-02
Arf1 pathway	9.78E-03	HIF-1-alpha transcription factor network	2.07E-02
RhoA signaling pathway	1.45E-02	ATR signaling pathway	2.12E-02

Supplementary Table 25. Top-ranked altered pathways (NCI-Nature pathway database) of untreated PGCCs versus non-PGCCs of Vari068 breast cancer cells.

Vario68	P-value
**PLK1 signaling events**	6.70E-08
E2F transcription factor network	2.28E-05
ATR signaling pathway	2.76E-05
**Aurora B signaling**	2.76E-05
**FOXM1 transcription factor network**	3.50E-05
p73 transcription factor network	5.80E-05
Validated targets of C-MYC transcriptional activation	2.61E-04
Direct p53 effectors	2.45E-03
IGF1 pathway	3.11E-03
FoxO family signaling	3.60E-03
Notch signaling pathway	3.84E-03
E-cadherin signaling in the nascent adherens junction	4.14E-03
Validated nuclear estrogen receptor alpha network	6.64E-03
Regulation of retinoblastoma protein	6.64E-03
Validated transcriptional targets of TAp63 isoforms	6.69E-03

Supplementary Table 26 Sequences of primers for qRT-PCR

hFTH1 qPCR F (CCTCCTACGTTTACCTGTCCATGTCTTAC)

hFTH1 qPCR R (ACCTCGTTGGTTCTGCAGCTTC)

hCDC20 qPCR F (GACCACTCCTAGCAAACCTGG)

hCDC20 qPCR R (GGGCGTCTGGCTGTTTTCA)

hCCNB1 qPCR F (AATAAGGCGAAGATCAACATGGC)

hCCNB1 qPCR R (TTTGTTACCAATGTCCCCAAGAG)

hActin qPCR F1 (CATGTACGTTGCTATCCAGGC)

hActin qPCR R1 (CTCCTTAATGTCACGCACGAT)

Supplementary Table 27 IC50s for rescue and sensitize cells to ferroptosis by altering ROS.

Cell lines	IC50s (μM)		
	RSL3	RSL3 with Rotenone	RSL3 with GSK2795039
Vari 068	0.18	0.12	0.81
SUM 159	0.32	0.12	1.49
BT474	0.23	0.1	2.26
SKBR3	0.31	0.05	5.98
Mean	0.26	0.098	2.64
P Value		0.014	0.0377

Supplementary Table 28 IC50s for rescue and sensitize PGCCs to ferroptosis by altering ROS.

Cell lines	IC50s (μM)		
	RSL3	RSL3 with Rotenone	RSL3 with GSK2795039
Vari 068	0.87	0.95	5.75
SUM 159	0.51	0.48	2.78
BT474	0.18	0.03	13.64
SKBR3	0.49	0.13	7.24
Mean	0.51	0.40	7.35
P Value		0.3094	0.0426

Supplementary Table 29 IC50s for rescue and sensitize non-PGCCs to ferroptosis by altering ROS.

Cell lines	IC50s (μM)		
	RSL3	RSL3 with Rotenone	RSL3 with GSK2795039
Vari 068	0.37	0.16	1.05
SUM 159	0.27	0.23	3.63
BT474	0.23	0.03	12.27
SKBR3	0.71	0.24	15.11
Mean	0.40	0.17	8.02
P Value		0.022	0.0382
