# Supplementary material for: Single-cell morphological and transcriptome analysis unveil inhibitors of polyploid giant breast cancer cells in vitro
Source: Commun Biol. 2023 Dec 21;6:1301. doi: 10.1038/s42003-023-05674-5 (PMC10739852; doi:10.1038/s42003-023-05674-5)
Supplement: Supplementary file 2 — Description of Additional Supplementary Files [file 42003_2023_5674_MOESM2_ESM.pdf]

Description of Additional Supplementary Files

File Name: Supplementary Movie 1

Description: Docetaxel treatment of SUM159 cells

File Name: Supplementary Data 1

Description: The list of compounds in the library.

File Name: Supplementary Data 2

Description: Statistical results of compounds cytotoxic effects screening for non-PGCCs.

File Name: Supplementary Data 3

Description: Statistical results of compounds cytotoxic effects screening for PGCCs.

File Name: Supplementary Data 4

Description: Statistical results of anti-PGCC inhibitors screening for non-PGCCs.

File Name: Supplementary Data 5

Description: Statistical results of anti-PGCC inhibitors screening for PGCCs.

File Name: Supplementary Data 6

Description: Source data behind the graphs in the figures.

File Name: Supplementary Software 1

Description: Code for single-cell morphological analysis.
